# Identification of three bacterial species associated with increased appendicular lean mass: the HUNT study

**DOI:** 10.1038/s41467-023-37978-9

**Published:** 2023-04-20

**Authors:** Louise Grahnemo, Maria Nethander, Eivind Coward, Maiken Elvestad Gabrielsen, Satya Sree, Jean-Marc Billod, Klara Sjögren, Lars Engstrand, Koen F. Dekkers, Tove Fall, Arnulf Langhammer, Kristian Hveem, Claes Ohlsson

**Affiliations:** 1 Department of Internal Medicine and Clinical Nutrition, Institute of Medicine, Sahlgrenska Osteoporosis Centre, Centre for Bone and Arthritis Research at the Sahlgrenska Academy, University of Gothenburg, Gothenburg, Sweden; 2 Bioinformatics Core Facility, Sahlgrenska Academy, University of Gothenburg, Gothenburg, Sweden; 3 K.G. Jebsen Center for Genetic Epidemiology, Department of Public Health and Nursing, NTNU, Norwegian University of Science and Technology, Trondheim, Norway; 4 Bio-Me, Oslo Science Park, Gaustadalléen 21, N-0349 Oslo, Norway; 5 Department of Microbiology, Tumor and Cell Biology, Centre for Translational Microbiome Research, Karolinska Institutet, Karolinska Hospital, Biomedicum A8, Solnavägen 9, 171 65 Stockholm, Sweden; 6 Department of Medical Sciences, Molecular Epidemiology and Science for Life Laboratory, Uppsala University, Uppsala, Sweden; 7 HUNT Research Centre, Department of Public Health and Nursing, NTNU, Norwegian University of Science and Technology, Levanger, Norway; 8 Levanger Hospital, Nord-Trøndelag Hospital Trust, Levanger, Norway; 9 Region Västra Götaland, Sahlgrenska University Hospital, Department of Drug Treatment, Gothenburg, Sweden

**Keywords:** Biomarkers, Microbiology, Osteoporosis

## Abstract

Appendicular lean mass (ALM) associates with mobility and bone mineral density (BMD). While associations between gut microbiota composition and ALM have been reported, previous studies rely on relatively small sample sizes. Here, we determine the associations between prevalent gut microbes and ALM in large discovery and replication cohorts with information on relevant confounders within the population-based Norwegian HUNT cohort (n = 5196, including women and men). We show that the presence of three bacterial species – *Coprococcus comes*, *Dorea longicatena*, and *Eubacterium ventriosum* – are reproducibly associated with higher ALM. When combined into an anabolic species count, participants with all three anabolic species have 0.80 kg higher ALM than those without any. In an exploratory analysis, the anabolic species count is positively associated with femoral neck and total hip BMD. We conclude that the anabolic species count may be used as a marker of ALM and BMD. The therapeutic potential of these anabolic species to prevent sarcopenia and osteoporosis needs to be determined.

## Abstract (web summary)

Here, the authors employ data from the population-based Norwegian HUNT cohort (n = 5196, including women and men) to associate the presence of three gut microbial species – *Coprococcus comes*, *Dorea longicatena*, and *Eubacterium ventriosum* – with higher lean mass.

## Introduction

Appendicular lean mass (ALM), measured by dual-energy x-ray absorptiometry (DXA) or bioelectrical impedance analysis (BIA), is associated with mobility, bone mineral density (BMD), and metabolic function^1–4^. With aging, there is a loss of skeletal muscle mass, and a concurrent increase in fatty infiltration and fibrosis of muscle. This loss of muscle mass may reach a critical point at which functional impairment and even disability occurs^4–6^. In mice, the gut microbiota (GM) has been shown to regulate muscle mass. The skeletal muscle mass and function were reduced in germ-free mice compared with conventionally-raised mice with GM, while the muscle mass was restored in the germ-free mice following GM transplantation^7,8^. Similarly, muscle mass and function were reduced in antibiotic-treated mice and restored following the recovery of the GM^9–11^.

In humans, it is unknown whether the GM regulates ALM or muscle mass and function. Most previous human association studies have quantified microbes at the genus level^12–18^, thus missing important species information. Only a few small studies (*n* < 500 subjects) have quantified GM composition at the species level and evaluated associations with lean mass^12,15^. The results of association studies between GM and lean mass are inconsistent, most likely because the studies, in general, have been underpowered and without proper control over confounders^19–21^. Confounders – age, sex, diet, smoking, medications, comorbidities, alcohol intake, and bowel movement quality – can influence the GM composition and hence interfere with GM associations; therefore, adjusting for confounders is essential^20,21^. Another weakness with previous human association studies is that they have not explored possible reverse causality whereby lean mass influences the GM composition.

The aim of the present study was to identify gut microbial species that are reproducibly associated with ALM, as measured by BIA. It should be emphasized that ALM, as measured by BIA, does not only include muscle but also water, skin, and fibrotic and connective tissue^22^. Thus, it is unclear if possible findings from the present study regarding ALM can be translated to muscle per se. We established a large population-based discovery (*n* = 2866) and replication cohort (*n* = 2330) with extensive information on relevant confounders within the population-based Norwegian HUNT-cohort (combined *n* = 5196). The abundance of 50 prevalent and well-characterized gut microbes was determined using a quantitative PCR method validated in silico and in vitro;^23,24^ species significantly associated with lean mass were also validated against metagenome sequencing. The present cohort was well-powered for the primary outcome ALM. However, as ALM associates with BMD^25,26^ and GM is known to regulate bone mass^27–30^, we also performed a less powered, explorative, sub-analysis of BMD measured by DXA (*n* = 1028 subjects). To this end, we determined if one exposure, the main identified ALM-associated feature, was associated also with BMD.

In this work, we identify three anabolic gut microbial species that, when combined into an anabolic species count, are directly associated with both ALM and BMD. Future studies are warranted to determine their possible therapeutic potential to prevent sarcopenia and osteoporosis.

## Results

### The presence of three bacterial species was associated with high ALM

**Fig. 1:**
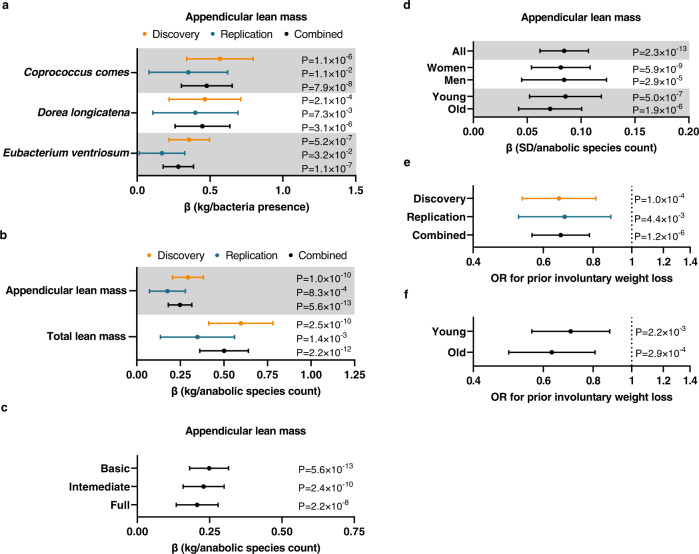
Identification of three bacteria that associated with appendicular lean mass (ALM). **a** Associations between the presence of *Coprococcus comes*, *Dorea longicatena*, and *Eubacterium ventriosum* with ALM (adjusted for age, gender, body fat mass, and cohort- and batch-specific covariates – basic model) in the discovery (*n* = 2866), replication (*n* = 2330), and combined cohorts (*n* = 5196). Forest plot of the point estimates (linear regression β coefficient; β in kg for the presence of bacteria) and 95% confidence intervals [CIs]. **b** Associations between the anabolic species count with ALM and total lean mass (basic model) in the discovery (*n* = 2866), replication (*n* = 2330), and combined cohorts (*n* = 5196). Forest plot of the point estimates (linear regression β coefficient; β in kg of lean mass per anabolic species count) and 95% CIs. **c** Associations between the anabolic species count and ALM in the basic model (*n* = 5196), intermediate model (i.e., basic model plus adjustments for chronic disease, smoking status, stool consistency, and use of prescription medication, *n* = 4773), and the full model (i.e., intermediate model plus adjustments for alcohol use and detailed information on dietary intake, *n* = 4535). Forest plot of the point estimates (linear regression β coefficient; β values in kg of lean mass per anabolic species count) and 95% CIs. **d** Stratified analyses on gender (*n* = 3317 women, *n* = 1879 men) and age (above [*n* = 2605] vs below [*n* = 2591] median age of 59.3 years) for the association between anabolic species count and ALM (combined *n* = 5196). Forest plot of the point estimates (linear regression β coefficient; β values in standard deviation [SD] of lean mass per increase in anabolic species count) and 95% CIs. **e**, **f** Associations between prior involuntary weight loss and anabolic species count in (**e**) the discovery (*n* = 2746), replication (*n* = 2271), combined cohorts (*n* = 5017), and in (**f**) young (*n* = 2540) and old (*n* = 2477) participants (above/below the median at 59.3 years). Forest plot of the odds ratios (OR) and 95% CIs from logistic regressions. All tests were two-sided. n refers to number of study participants. Source data are provided as a Source Data file.

The present study included participants from the Norwegian population-based HUNT cohort (Supplementary Fig. 1; Supplementary Table 1). The main aim was to identify specific microbes at the species level that were reproducibly associated with ALM. Analyses were adjusted for age, gender, height, and body fat mass (basic model). Using microbial relative abundances as binary data (absence/presence), linear regression analyses showed that 5 microbes were associated with ALM following Bonferroni correction in the discovery cohort, while the use of data in quartiles resulted in 4 associated microbes (Supplementary Table 2). For these microbes to be eligible for replication, they also needed to be nominally significantly associated with ALM without fat mass adjustment. Three microbes, *Dorea longicatena (D. longicatena)*, *Coprococcus comes (C. comes)*, and *Eubacterium ventriosum (E. ventriosum)*, fulfilled the criteria for replication using binary and quartile microbial data (Supplementary Table 2–3). The association with ALM was replicated for all three microbes when using binary data (Fig. 1a; Supplementary Table 4), and for *D. longicatena* when using quartile data (Fig. 1a; Supplementary Table 5). As some quantitative information is lost when data are truncated into quartiles, we also performed additional sensitivity analyses in which microbial data were centered log-ratio (CLR) transformed, demonstrating that all three anabolic species were associated with ALM in the discovery cohort (Supplementary Table 6) and that both *D. longicatena* and *C. comes* were associated with ALM also in the replication cohort (Supplementary Table 6). As the most robust replication for all three species was observed for binary data, the presence/absence (binary data) of *D. longicatena, C. comes* and *E. ventriosum* were used for further analyses.

In the combined cohorts, ALM was increased in individuals with the presence of *C. comes* (+0.48 kg), *D. longicatena* (+0.45 kg), and *E. ventriosum* (+0.28 kg, Fig. 1a; Supplementary Table 7). Similar to ALM, total lean mass was also increased in individuals with the presence of *C. comes* (+0.97 kg), *D. longicatena* (+0.91 kg), and *E. ventriosum* (+0.57 kg, Supplementary Table 7). These associations were only marginally attenuated after adjustment for multiple confounders (chronic diseases, medication, smoking status, and stool consistency, i.e., the intermediate model) and after further adjustments for alcohol and dietary intake (i.e., the full model, Supplementary Table 7). Stratified analyses showed similar effect sizes in men and women and in old and young subjects (above/below median age) for the three anabolic bacteria (Supplementary Table 8).

### An anabolic species count was robustly directly associated with ALM

Using Pearson’s correlation of binary data (absence/presence), we found that all three anabolic bacterial species identified in this study correlated modestly, explaining less than 13% of the variation in pairwise comparisons (*C. comes* – *D. longicatena* r^2^ = 0.127, *C. comes* – *E. ventriosum* r^2^ = 0.044, and *D. longicatena* – *E. ventriosum* r^2^ = 0.051). We next included all three species in the same linear regression models. Although the effect sizes of the individual species were slightly reduced in the combined model with multiple bacteria, all three species remained significantly directly associated with ALM and total lean mass (Supplementary Table 9). As these analyses suggest that *C. comes*, *D. longicatena*, and *E. ventriosum* provide independent information, we combined the data on these three anabolic bacterial species into an index of number of anabolic bacterial species present in the study subjects (ranging from 0 to 3). Using this anabolic species count, we found that individuals with all three species had 0.80 kg higher ALM (P for trend = 5.6 × 10^−13^) and 1.60 kg higher total lean mass (*P* for trend = 2.2 × 10^−12^) than individuals without any of these (Table 1). The associations between the anabolic species count and ALM were found for the discovery, replication, and combined cohorts (Fig. 1b). The association between the anabolic species count and ALM was only slightly reduced when adjusting for multiple relevant covariates, and the association was similar in men and women and in young and old individuals (Fig. 1c, d). In sensitivity analyses, we observed that the association between the anabolic species count and ALM remained unchanged when removing individuals that did not live in Norway at 1 year of age (Supplementary Table 10).

**Table 1:** Appendicular and total lean mass for individuals with different number of anabolic species present

	No. of anabolic species present	Estimated marginal mean (±95% CI)	P for trend
ALM			5.6E-13
	0	21.8 (21.4–22.2)	
	1	22.1 (21.8–22.4)	
	2	22.3 (22.0–22.6)	
	3	22.6 (22.3–22.9)	
Total lean mass			2.2E-12
	0	49.5 (48.7–50.3)	
	1	50.1 (49.4–50.8)	
	2	50.6 (50.0–51.2)	
	3	51.1 (50.5–51.7)	

Appendicular lean mass (ALM) and total lean mass for individuals with the presence of 0, 1, 2, or 3 anabolic species (combining the information on the presence of *Coprococcus comes*, *Dorea longicatena*, and *Eubacterium ventriosum*), presented as estimated marginal means (±95% confidence intervalls [CIs]). Linear regression between the anabolic species count as exposure with ALM and total lean mass as the outcome using the basic model in the combined cohorts (*n* = 5196) were used to obtain P for trend. Tests are 2-sided.

A sensitivity analysis showed that removal of the adjustments for batch-specific covariates did not affect the main finding in the present study, the robust association between the anabolic species count and ALM (with adjustment for batch-specific covariates: β = 0.25 95% CI 0.18–0.32, *P* = 5.6 × 10^−13^; without adjustment for batch-specific covariates: β = 0.25, 95% CI 0.19–0.32, *P* = 2.0 × 10^−13^). In addition, the effect estimate of the association between the anabolic species count and ALM for the combined data set was very similar when the results from the three batches were combined using an inverse variance weighted meta-analysis (β = 0.25, 95% CI 0.18–0.32, *P* = 1.9 × 10^−13^) instead of pooling.

Next, we evaluated if the anabolic species count was associated with involuntary weight loss (>5 kg the past six months). Logistic regression analyses showed that the anabolic species count was negatively associated with involuntary weight loss in both the discovery and replication cohorts (Fig. 1e) and both in young and old subjects when evaluated age-stratified (Fig. 1f).

### An anabolic species count was directly associated with BMD

As low ALM is a risk factor for osteoporosis, we followed up on the finding that the anabolic species count was associated with the primary outcome ALM by testing whether this exposure also associates with BMD. In the subset of participants with available BMD data (*n* = 1028), less powered, explorative sub-analyses showed that the anabolic species count was directly associated with femoral neck BMD (β = 0.013 g/cm^2^ per increase in anabolic species count [95% CI 0.0035–0.022], *P* = 0.007) and total hip BMD (β = 0.013 g/cm^2^ [95% CI 0.0032–0.022], *P* = 0.009).

### No evidence of a causal association for genetically determined ALM on the anabolic species count

**Fig. 2:**
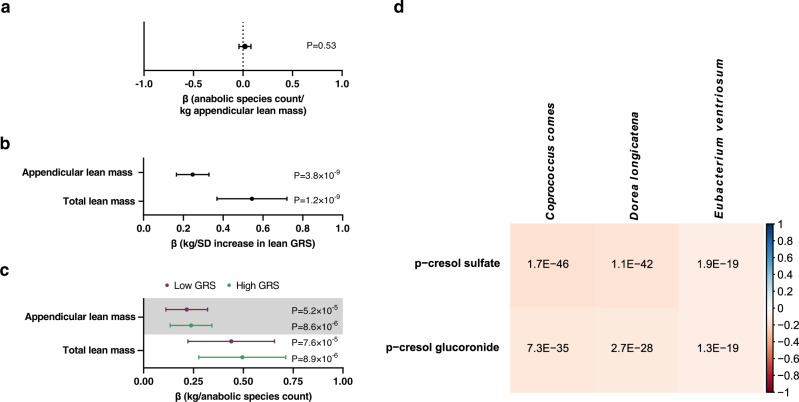
Studies on the underlying nature of the associations between three anabolic species and appendicular lean mass (ALM). **a** Two-sample Mendelian randomization (MR) analysis using genetically determined ALM as the exposure and the anabolic species count as the outcome. Forest plot of the point estimate (β values as change in anabolic bacteria count/kg increase in genetically determined ALM) and 95% confidence intervals (CIs) from a fixed effect inverse variance weighted MR (*n* = 4002). **b** Associations (gender- and age-adjusted) between ALM genetic risk score (GRS) and ALM and total lean mass in HUNT. Forest plot of the point estimates (linear regression β coefficient; β values in kg lean mass/standard deviation [SD] increase in ALM GRS) and 95% CIs (*n* = 4002). **c** Stratified evaluation of associations between anabolic species count and lean mass in subjects with low and high ALM GRS (divided by the median). Forest plot of the point estimates (linear regression β coefficient; β values in kg of lean mass per anabolic species count) and 95% CIs (*n* = 4002). **d** Partial Spearman’s rank correlations between bacterial species relative abundance and circulating levels of metabolites, adjusted for age, sex, place of birth, study site, microbial DNA extraction plate, and metabolomics delivery batch, in the dataset by Dekkers et al.^32^ (*n* = 8583). All tests are 2-sided. n refers to number of study participants.

A strength of the present study is that genetic information was available for most of the subjects with available GM analyses, enabling Mendelian randomization analysis to test for possible reverse causality. We performed a two-sample Mendelian randomization analysis using genetically determined ALM as the exposure (using genetic instruments from a previous ALM GWAS^31^) and anabolic species count as outcome. The analysis showed no evidence of reverse causality of ALM on the anabolic species count (Fig. 2a; Supplementary Data 1). We also performed a sensitivity analysis only including the large, randomly selected discovery cohort, not requiring batch-specific adjustments, for the outcome association analyses used in the MR. This sensitivity analysis showed similar results of no evidence of reverse causality of ALM on the anabolic species count (inverse variance weighted MR results when using both the discovery and replication cohorts: β = 0.02, 95% CI −0.04 to 0.08, *p* = 0.53, *n* = 4002, Fig. 2a; MR results when using only the discovery cohort: β = 0.00, 95% CI −0.08 to 0.08, *P* = 0.99, *n* = 2292).

Next, we developed a weighted ALM genetic risk score (GRS) based on a previous ALM GWAS^31^. As expected, this ALM GRS was associated with both ALM and total lean mass in the HUNT cohort (Fig. 2b). However, this GRS was not associated with the anabolic species count in HUNT (β = 0.001 anabolic species count/SD ALM GRS [95% CI −0.022–0.025], *P* = 0.91), supporting the notion that genetically determined ALM does not influence the anabolic species count. Furthermore, the anabolic species count was associated with lean mass both in individuals with low and high ALM GRS (divided by the median) (Fig. 2c).

### The three identified anabolic species are associated with circulating metabolites

GM may affect its host partly by affecting the levels of circulating metabolites. To identify metabolites correlated with the anabolic bacteria identified in the present study, we used a unique data set on the correlation between the abundance of gut microbial species, determined by metagenome sequencing, and circulating levels of more than 1000 metabolites^32^. When evaluating the top four positive and negative correlations for the three identified anabolic bacterial species with circulating metabolites (Fig. 2d; Supplementary Table 11), we observed that all three bacteria were consistently and strongly negatively correlated with p-cresol sulfate and the highly related p-cresol glucuronide. These associations may inform on underlying mechanisms for our observed associations between bacteria and ALM.

## Discussion

In humans, the associations between gut microbiota composition and ALM is poorly investigated. Using the largest GM data set thus far, we identified three bacterial species reproducibly associated with ALM also after adjustment for multiple relevant covariates, individually and when combined into an anabolic species count. In explorative analyses, the anabolic species count – the main identified feature associated with our primary outcome – was inversely associated with involuntary weight loss, and in a minor, less powered subset of the cohort, the anabolic species count was also positively associated with BMD. We found no evidence of reverse causality in which genetically determined ALM influences the anabolic species count. The three anabolic species were consistently associated with two circulating metabolites that may mediate some of the potential effect of the bacterial species on ALM.

Some small human studies have tested for associations between GM taxa and lean mass, with varied results^12–18^. The inconsistent results may be caused by the small sample size and often lack of proper adjustments. Furthermore, as most studies quantified GM using 16 S sequencing that do not provide detailed species level information, the observed associations are mostly at the genus level. Within a genus, the different species may affect lean mass differently; therefore, important species associations may be hidden at the genus level. Our study overcomes these problems due to its large sample size, species level information, and the possibility to replicate and adjust for relevant confounders. Additionally, we could test for possible reverse causality in which ALM influences the GM. Our two-sample Mendelian randomization analysis showed no evidence for reverse causality.

In line with our data, the genera *Dorea*, to which *D. longicatena* belongs to, and *Coprococcus*, to which *C. comes* belongs to, were positively associated with lean mass in a small study of older persons with HIV (*n* = 14)^33^. The genus *Coprococcus* has also been associated with higher lean mass in premenopausal women (*n* = 40)^18^. Similarly, the genus *Eubacterium*, to which *E. ventriosum* belongs to, was decreased in patients with frailty and/or sarcopenia, diseases associated with low lean mass, compared with controls (*n* = 35 and 87, respectively)^34,35^. As these studies were very small and only provided data at the genus level, the previous line of evidence is weak. However, we provide species level data, replication of our findings, and a combined cohort that is over 50 times larger than these previous studies – strengthening the evidence immensely.

Gut microbes may mediate some of their effects trough production of short-chain fatty acids (SCFA). These bacterial metabolites have been shown to increase muscle mass in mice by inhibiting muscle atrophy^7,36^. In humans, butyrate metabolism pathways and genetically determined synthesis of butyrate have been associated with lean mass^12,37^. Interestingly, all the three anabolic bacteria identified in the present study have been shown to produce SCFAs: *C. comes* produces acetate and butyrate, *D. longicatena* produces acetate and formate, and *E. ventriosum* produces butyrate^38–40^. These SCFAs may mediate some of the potential anabolic effect of the identified anabolic bacteria on ALM.

Apart from SCFA, GM also produces other metabolites that may, in part, explain an anabolic effect of *C. comes*, *D. longicatena*, and *E. ventriosum*. In a separate cohort, we found that the three identified anabolic bacteria were strongly and negatively associated with p-cresol sulfate and p-cresol glucuronide. An important functional study evaluated the effect of p-cresol sulfate, an uremic toxin of bacterial origin, on muscle tissue in mice^41^. Administration of p-cresol sulfate in that study resulted in substantial ectopic lipid accumulation in skeletal muscle in mice^41^. Furthermore, p-cresol sulfate altered the insulin signaling in myocytes, a likely mechanism for the observed insulin resistance in the p-cresol-treated mice^41^. This previous mouse treatment study demonstrates that p-cresol sulfate exerts deleterious effects in muscle tissue, affecting muscle composition and metabolic functionality. As BIA, used for the body composition measurements in the HUNT cohort, is used to identify lean mass, not including fat mass, a possible increase in relative fat content in muscle would be reflected by an apparent reduced relative lean mass. This claim is coherent with the findings from the present human association study in which all three anabolic species are associated with increased ALM and decreased circulating p-cresol sulphate in humans. In contrast, the related p-cresol glucuronide did not exert any deleterious effect in muscle when given to mice^41^, suggesting that p-cresol sulfate but not p-cresol glucuronide is biologically active^42,43^. Thus, for p-cresol sulfate, there is direct functional data to support an effect on muscle tissue. Although the anabolic species are correlated to p-cresol sulfate and p-cresol glucuronide, we cannot exclude the possibility that other bacterial species are more strongly correlated with the levels of these metabolites and that the identified anabolic species might merely covary with these other bacterial species. Future studies are warranted to determine if any of the three identified anabolic species regulate ALM via SCFAs and/or the metabolite p-cresol sulfate.

Similar as in our previous study identifying genetic determinants of appendicular lean mass^5^, we adjusted for body fat and height but not weight. There are other options for adjustment, but we chose the same approach as in our previous co-authored study searching for genetic associations with appendicular lean mass^5^. Adjustment for fat mass and not weight may enhance the likelihood of identifying determinants associated specifically with lean mass^5^.

A limitation with the current study is that we did not quantify the GM using metagenome sequencing; therefore, we have no direct information on possible functional pathways that may mediate effects of the three anabolic bacteria on ALM in the HUNT cohort or any information on less common and characterized microbial species. However, analyses of a large number of microbial species derived from metagenome sequencing would, in general, have been underpowered in the present setting including 2866 subjects in the discovery cohort^44^. Future large-scale studies evaluated using metagenome sequencing or meta-analyses of several medium-sized cohorts are needed to identify less abundant microbial species associated with ALM. Another limitation is that the participants are mainly of Norwegian ancestry. Future studies are, therefore, necessary to test whether our findings also apply to those of other ethnicities. In addition, it is a limitation that we, besides measurements of ALM using BIA, did not have state-of-the-art measurements of muscle mass using the D_3_-Creatine dilution method^22^ in the HUNT cohort. ALM analyzed by BIA, as in the present study, does not only include muscle but also water, skin, and fibrotic and connective tissue^22^. Thus, it is unclear if the findings from the present study regarding ALM can be translated to muscle per se. Future studies are warranted to determine the associations between gut microbes and muscle mass, determined by a valid method. Furthermore, we only tested for reverse causality, in which lean mass influences anabolic species, as currently available genetic instruments are too weak to test for causality, in which presence of anabolic bacteria influences lean mass. Stronger genetic instruments for bacterial species are required for future testing of causality.

A recent study including the MrOS and FHS cohorts emphasized that large cohorts (*n* > 6000) are required to identify GM features associated with bone-related parameters when analyzing GM by the 16 S method^44^. Therefore, the present subset of the HUNT cohort (*n* = 1028) with available measurements of microbial species and BMD is clearly underpowered for broad analyses of associations between bacterial species and BMD. However, in a less powered, exploratory sub analysis, we tested whether one exposure, the anabolic species count that was identified as the main ALM-associated feature in the present study, also associates with BMD. We found that the anabolic species count was associated with increased BMD, but our BMD analysis was explorative and included a modest data set of subjects with available BMD data. Large well-powered data sets with information on GM composition and BMD need to be established for well-powered, broad analyses of the association between GM composition and BMD^44^.

In conclusion, we identified three anabolic gut microbial species that, when combined into an anabolic species count, are directly associated with both ALM and BMD. Future studies are warranted to determine their possible therapeutic potential to prevent sarcopenia and osteoporosis.

## Methods

### Ethical statement

The study was approved by the Regional Committee for Medical and Health Research Ethics in Central Norway (reference number 28052). All participants provided written informed consent. The participants did not receive any compensation.

### Sample size selection

Previous studies evaluating the association between gut microbes at the species level and lean mass have included 482 subjects or less and have not included replication or detailed adjustments for multiple relevant confounders^12,15^. To identify possible reproducible associations with the primary outcome appendicular lean mass, we established a large discovery cohort (*n* = 2866) and one replication cohort (*n* = 2330) with in total 5196 participants.

### Study participants

The Trøndelag Health Study (HUNT) is a comprehensive population-based study that has collected data in four surveys in the region of Nord-Trøndelag, Norway from 1984 to 2019 (HUNT1–4). Our cross-sectional study includes participants in HUNT4 (2017–19)^45,46^. All people living in the county of Nord-Trøndelag (later the two counties South and North Trøndelag have been fused into one county, Trøndelag), Mid-Norway, that would pass the age of 20 during the period the field stations were in their municipality were eligible to participate in HUNT4. Eligible participants, identified through the Norwegian National Population Register, were invited to HUNT4 by the HUNT research center, and if needed, they were reminded once. Written informed consent for the present study including feces samples was obtained at the HUNT4 baseline visit before kits for feces sampling was given to the participants. In total, 13,268 of 55,561 subjects (23.9%) submitted stool samples and answered a questionnaire on stool consistency.

The present study included participants with ALM data and stool microbiota data passing a quality check, divided into a discovery cohort and one replication cohort (Supplementary Fig. 1, Supplementary Table 1). The discovery cohort consisted of 2866 randomly selected participants (mean age 60.3 ± 13.9 standard deviation [SD], 59.4% women), with qPCR analyses of microbial species in one batch (batch 1). The replication cohort consisted of 2330 participants (mean age 53.8 ± 14.0 SD, 69.3% women), analyzed in two batches (batch 2 and 3). Batch 2 consisted of 949 selected healthy participants (see below for definition of healthy participants) and 44 randomly selected participants. Batch 3 consisted of 1337 participants that were selected migraine cases (*n* = 741) or controls (*n* = 596) in a migraine sub-study (Supplementary Fig. 1; Supplementary Table 1). The representativity of the participants are described in Supplementary Note 1 and Supplementary Table 12.

We have adjusted for (1) analysis batch, (2) whether participants of batch 2 were a selected healthy subject, and (3) whether participants of batch 3 were a selected migraine case. As subjects in batch 2 cannot be a selected migraine case, we performed sensitivity analyses that all support the notion that the batch-specific adjustments did not bias the effect estimate of the main finding of the present study (see results).

The healthy participants of batch 2 were individuals that (1) valued their current health as good or very good, (2) did not take medication for asthma, anxiety, allergy, depression, thyroid problems, high cholesterol, high blood pressure, chronic obstructive pulmonary disease, or any other prescription medication, and (3) did not have angina pectoris, myocardial infarction, atrial fibrillation, stroke, asthma, chronic obstructive pulmonary disease, diabetes, hypothyroidism, hyperthyroidism, cancer, migraine, psoriasis, renal disease, rheumatoid arthritis, spondylarthritis, gout, or mental problems.

### Collection of questionnaire data

Collection of questionnaire data have previously been described in detail^24,45,46^. In brief, HUNT4 participants responded to questionnaires regarding their gender, health (Chronic disease Yes/No was the answer to the question: Do you suffer from longstanding [at least 1 year] illness or injury of a physical or psychological nature that impairs your functioning in your daily life?), medication, smoking, and country of residence at 1 year of age. They also answered questions regarding last year’s weekly intake of fruit/berries, vegetables, red meat (beef, pork, lamb, game), white meat (chicken, turkey), processed meat (e.g., minced meat, sausage), low-fat fish (e.g., cod, pollock), and high-fat fish (e.g., salmon, trout, herring, mackerel, haddock). Participants also answered questions regarding last year’s alcohol frequency of 6 or more glasses in one sitting. Furthermore, study participants were asked to score their stool sample according to the Bristol stool scale^47^. This scale is numbered 1–7, with one being the hardest type of stool and seven being completely liquid. For statistical analyses, the Bristol stool scale data were recoded into 3 categories: Bristol stool scale 1–2 was categorized as hard stool that was coded as 1, Bristol stool scale 3–4 were categorized as normal stool and coded as 2, and Bristol stool scale 5–7 were categorized as loose stool and coded as 3^21^.

### Anthropometric and body composition measurements

Anthropometric (height, weight) and body composition (body fat mass, ALM and total lean mass) data were obtained by direct segmental measurement multi-frequency BIA (InBody770®). The InBody770® device measures impedance in five separate compartments (left and right arm, left and right leg, trunk) by using 8 tactile electrodes (anterior and posterior part of each foot and thumb and palm of each hand) to send currents through the body at 6 different frequencies (1, 5, 50, 250, 500, and 1000 kHz) and measure the resulting voltage. Lean mass was defined as fat-free mass. During analysis, the participants were barefoot. Prior involuntary weight loss of >5 kg the past six months (yes/no) was evaluated using a questionnaire.

### Bone densitometry

Bone densitometry of the left hip (total and femoral neck) was performed by trained staff in four municipalities by two different DXA machines (Hologic and Lunar Prodigy). Regions of interest were systematically evaluated. To be able to use total hip and femoral neck DXA measurements performed with equipment from two different manufactures (Hologic, *n* = 461 and Lunar Prodigy, *n* = 582), standardized BMD was calculated for these bone sites as previously described^48,49^. Furthermore, to minimize the possible confounding effect of using two different DXA machines, DXA type (Hologic or Lunar Prodigy) was included as a covariate in all regression analyses with total hip and femoral neck BMDs as dependent factors. The BMD regression analyses were further adjusted for age, gender, cohort- and batch-specific covariates, scanner location, and body mass index.

### Gut microbiota analysis

Stool collection, DNA extraction, and microbiome analysis were performed as previously described^23,24^. In brief, stool samples were collected on filter papers in the homes of the participants and then sent to the HUNT biobank for storage at –80 °C. Three 6 mm discs were punched out from each filter card into an allocated well on MagMAX™ 96 Deep Well Plates (Thermo Fisher Scientific, Waltham, USA). After bead-beating to disrupt microbial cell walls, DNA was isolated using the Microbiome MagMAX Ultra kit (Thermo Fisher Scientific) following the manufacturer’s recommendations on KingFisher™ Flex (Thermo Fisher Scientific). The amount of DNA was quantified using Quant-iT™ PicoGreen™ dsDNA Reagent (Thermo Fisher Scientific).

Next, the composition of the gut microbiota was analyzed by a validated quantitative PCR method (Bio-Me’s Precision Microbiome Profiling [PMP™]), based on TaqMan™ technology on OpenArray® format (Thermo Fisher Scientific), that targets 50 prevalent and well-characterized microbiota species of the human gut microbiome (Supplementary Table 2)^23,24^. Standard curves for the assays were generated by running reference materials quantified by fluorescence (Quant-iT™ PicoGreen™ dsDNA Reagent, Thermo Fisher Scientific). The absolute quantification of each target (number of genomic copies per µL) was interpolated from the standard curves. The relative abundance (%) was determined by dividing the absolute quantification of a target by the sum of the absolute quantification for all 50 evaluated targets in the sample.

All assays were validated in silico and in vitro for specificity and sensitivity, and in vitro for dynamic range and standard curve accuracy. For microbes with a relative abundance >0.3%, the mean CV was 8.4%. Due to large coefficients of variations for samples with very low measured levels, samples with undetectable levels as well as samples with relative abundance <0.005% were set to zero (both data on relative abundance and corresponding absolute levels). Samples were spiked in by a positive amplification control with a validity threshold set at quantitation cycle 23. Further, assay information is given in previous publications^23,24^ and in Supplementary Notes 2–3.

### ALM genetic risk score (GRS)

We defined a GRS based on previously identified single nucleotide polymorphisms (SNPs) associated with ALM^31^. Genotyping is described in Supplementary Note 4. For each individual, the GRS was defined as the weighted sum of SNP dosages, where SNP effects from the ALM genome-wide association study were used as weights. Out of the 4002 participants with genetic and lean mass information, *C. comes* was detected in 3635, *D. longicatena* was detected in 3678, and *E. ventriosum* was detected in 2371.

### Mendelian randomization

To evaluate a possible causal association of ALM on the anabolic species count, we performed two-sample Mendelian randomization (MR) analyses^50^. We used genetic instrument variables obtained from previously published genome-wide association studies on ALM^31^. Associations between each of the used genetic instruments and the anabolic species count were estimated with a linear mixed model adjusted for gender, age, cohort, and the first four principal components using the BOLT-LMM software (version 2.3.4) that also accounts for relatedness between samples. We used an inverse-variance weighted MR as the primary analysis. Possible heterogeneity was determined using Cochran’s Q statistic test. We then used the MR-Egger method as a sensitivity analysis to avoid possible uncontrolled pleiotropy^51^. In further sensitivity analyses, we used the penalized weighted median MR method and the weighted median MR methods. The analyses were conducted with the R-package MendelianRandomization (version 0.5.1)^52^.

### Correlations between metabolomics and metagenomics data

To identify metabolites correlated with the anabolic bacteria identified in the present study, we used a unique data set on the correlation between the abundance of microbial species determined by metagenome sequencing and circulating levels of more than 1000 metabolites^32^. In short, the data set contained data on 8583 participants (age 50–64) in the population-based Swedish CardioPulmonary bioImage Study (SCAPIS). Fecal samples were subjected to deep metagenome sequencing on an Illumina Novaseq 6000 system (Illumina, USA). Plasma metabolites were quantified using ultra-high-performance liquid chromatography (Metabolon Inc., Durham, NC, USA). Correlations were calculated for 1528 species and 1321 metabolites using partial Spearman’s rank test adjusted for age, sex, place of birth, study site, microbial DNA extraction plate, and metabolomics delivery batch^32^.

### Statistics and reproducibility

#### General

No statistical method was used to predetermine sample size. Following removal of individuals without appendicular lean mass data and stool microbiota data not passing a quality check, no data were excluded from the analyses.

Statistical analysis was done in R. To assess associations between the relative abundance of GM (with >10% relative abundance) and continuous parameters, we used linear regressions with the following adjustments: *the basic model* – age, gender, height, body fat mass, and cohort- and batch-specific covariates (when analyzing the replication cohort or the combined cohorts); *the intermediate model* – basic model plus adjustments for major confounders (chronic disease, smoking status, stool consistency, and use of prescription medication); and the *full model* – intermediate model plus adjustment for alcohol use and detailed information on dietary intake (food intake frequency of fruit/berries, vegetables, red meat, white meat, processed meat, low-fat fish, and high-fat fish). In a sensitivity analysis, we combined the results of the association between the anabolic species count and ALM in the different batches by performing a fixed effects inverse variance weighted meta-analysis using the R package metafor (version 3.8-1). The binary outcome involuntary weight loss was analyzed using logistic regression to show odds ratios. The R package emmeans (version 1.6.1) was used to obtain estimated marginal means for groups.

ALM and total lean mass were normally distributed. In general, the relative abundances of microbes were heavily skewed; therefore, the data for all microbes were divided into quartiles, with all the non-detectable values in the first quartile, or a binary variable indicating the presence or absence of microbes. As some information of quantitative data is lost when data are truncated into quartiles, we also performed additional analyses in which microbial data were CLR transformed using the R package microbiome (version 1.19.1)^53^, and all non-detectable values were given a fixed value. Using the binary variables for *C. comes*, *D. longicatena*, and *E. ventriosum*, we calculated an anabolic species count, indicating the number of anabolic species present (0–3). In general, the anabolic species count was used as a numerical exposure variable, except in Table 1. In Table 1, we used the anabolic species count as a categorical variable to be able to show estimated marginal means for individuals with 0, 1, 2, or 3 anabolic species. This table show that a linear relationship is likely between the anabolic species count and appendicular and total lean mass. To identify possible non-linearity in the associations, we also tested the addition of a squared term of the anabolic species count exposure in the model, but the squared term was not significant, arguing against a non-linear association.

In the initial screening for associations between gut microbial species (present in >10% of the participants) and the primary outcome ALM, we used the conservative Bonferroni correction to adjust for multiple comparisons; therefore, *p* ≤ 0.0012 (0.05/43) was considered statistically significant. For all other analyses, *p* ≤ 0.05 was considered statistically significant. All tests were 2-sided. Raw data were analyzed using R, version 4.1.1. Figures were processed in R or GraphPad Prism, version 9.1.0.

#### Handling of missing data

In the combined cohorts, there were 5196 individuals included in the basic model (available microbial and BIA data were required for inclusion in the present study, while there were 4773 individuals (423 missing one or more covariates) in the intermediate model and 4535 individuals (661 missing one or more covariates) in the full model. The intermediate and full models contained fewer individuals because there were individuals that missed data on one or more of the covariates included in these models, and we did not impute missing data. The numbers of individuals with missing data on the individual covariates were: 68 for chronic disease, 24 for smoking status, 328 for stool consistency, 27 for use of prescription medication, 44 for dietary intake of fruit and berries, 41 for dietary intake of vegetables, 64 for dietary intake of red meat, 72 for dietary intake of white meat, 69 for dietary intake of processed meat, 35 for dietary intake of low-fat fish, 35 for dietary intake of high-fat fish, and 114 for alcohol intake.

### Reporting summary

Further information on research design is available in the Nature Portfolio Reporting Summary linked to this article.

## Supplementary information

Supplementary Information

Description of Additional Supplementary Files

Supplementary Data 1

Reporting Summary

## Data Availability

Summary statistics are presented in the article. The individual participant data are available under restricted access for privacy issues. Researchers associated with Norwegian research institutes can apply for the use of HUNT data and samples with approval by the Regional Committee for Medical and Health Research Ethics. Researchers from other countries may apply if collaborating with a Norwegian Principal Investigator. Information for data access can be found at https://www.ntnu.edu/hunt/data. The HUNT variables are available for browsing on the HUNT databank at https://hunt-db.medisin.ntnu.no/hunt-db/. The Genome Reference Consortium Human genome (build 37) and revised Cambridge Reference Sequence of the human mitochondrial DNA (GenBank ID NC_012920) were obtained from http://genome.ucsc.edu. Source data are provided with this paper.

## Source data

Source Data
