# Supplementary material for: Identification of three bacterial species associated with increased appendicular lean mass: the HUNT study
Source: Nat Commun. 2023 Apr 20;14:2250. doi: 10.1038/s41467-023-37978-9 (PMC10119287; doi:10.1038/s41467-023-37978-9)
Supplement: Supplementary file 1 — Supplementary Information [file 41467_2023_37978_MOESM1_ESM.pdf]

SUPPLEMENTARY INFORMATION

Identification of three bacterial species associated with increased appendicular lean mass: the HUNT study

CONTENTS

Supplementary Notes	Page 2
Supplementary Figure 1	Page 4
Supplementary Table 1	Page 5
Supplementary Table 2	Page 6
Supplementary Table 3	Page 7
Supplementary Table 4	Page 8
Supplementary Table 5	Page 9
Supplementary Table 6	Page 10
Supplementary Table 7	Page 11
Supplementary Table 8	Page 12
Supplementary Table 9	Page 13
Supplementary Table 10	Page 14
Supplementary Table 11	Page 15
Supplementary Table 12	Page 16
Supplementary References	Page 17

Supplementary Note 1. Representativity

Comparison of the participants who agreed to participate in the HUNT4 baseline visit with those that did not participate is described in the recent cohort description article.[11] Age, gender, appendicular lean mass (ALM; main outcome), and total lean mass for the HUNT 4 participants that participated in the feces sub-study and those that did not participate is described in Supplementary Table 12. Subjects with available feces samples were slightly older and included a higher proportion of women than subjects without available feces samples. Comparisons between the participants in the present study with performed quantitative PCR (qPCR) analyses showed that the subjects in the discovery cohort were representative of the subjects with available feces samples. The subjects in the replication cohort were younger and included a higher proportion of women than the pool of subjects with feces samples available (Supplementary Table 12). Individuals in the combined cohorts with qPCR analyses and bone mineral density data included a high proportion of women. When stratifying on gender, ALM and total lean mass was similar in all subgroups (Supplementary Table 12).

Supplementary Note 2. Assay design

For the assays of the three anabolic species identified in this study, bioinformatic analyses identified conserved and unique target regions. ThermoFischer Scientific (Waltham, MA, USA) developed specific and unique primers and probes targeting these amplicon regions. The target region for *C. comes* was
AAGAATTGCTGCATTCCATTGTCAGGATATCTCCCAATGTCACATTTGGTTCTTTCACCTCGGTATT
CTTTTCCCCTTTGATATAAAGTATTTCATTTCCCGAAGACATCTGTATCCGTCCTTTCTTTTACCGGT
AGCGCCTGCCAGATCAGCGTTCCTTCACTTACCTGGTCTGTCCCCCGGACXTCATTATAATAGTAG
ATCATTCCGATCAGAACAGCTGTCAGCAGGGCACAAAGAACACAGCCCCAGATATATTTTTTTCTT
CTTTCCAATCGCATCCGCCTCCTTTGTGATCTGTTTCAGATAGGAAATAGTATCCCTGTTTTTCCAG
AAAACATACATCCTTTTATTCTTTTGCGTGTTCCCGCATGGTTTTCAGGATCTTTTTCTCCATCCGXG
ATACCTGCACCTGGGAAA. The target region for *E.ventriosum* was TTTATTCCTGTGATGAGAACCAGT
AATCTGCTGTATCCTTAAGGAATCCGTCTTTATCCATCTGTTCTAATGCTTTGTTAATAGCTTCCTG
AAGTTTTGCATTGTTTTTTCCAATAGCAATACCGAATTCTTCAGGTTCTGTCTTATCTGAAAATGCC
TTTTCATACTTGTCAGGATTCTTTGCTACGAAACCGTCAGCAACAGTACTATCAACTACTACTGCAT
CAATTTCACCATTTGTTAACTGTGTAAAGCAXTTTAATAACTTGTCATTAGCTGCTACTTCTACATT
AATAGTCTTTGTTGACTTGTATTCTGACATAAGCTGATCTGATGTTGTACCTTTCTGTACACCAACT
GATTTTCCGTCAAGATCCTTTAAGCTGTTAATCTTTAAGTCGCTACCCTTCTTAACTACTA. The target region for *D. longicatena* was TCCGTTCCGGTATATCCCGGATCACAGCCATGGTCTGCCGTAATC
ATCAACATATCGTCGTCCCGCATATTTGCGAGGAATTCCCCTAACTGTTCATCGAATCTCGTCAGTG
CTCCTGCATATCCTGCAATATCACGCCGGTGTCCATAGATCATATCCCCATCTACTAAGTTCACATA
GCAAAGTCCGTCGAACTCTTTCTTCTGTATCTCGATCGTCTTTTCCATATTCTTTTCATTTCCCTGAT
TCGGGTAGGTCTCCGTCACTCCCTGCCCTTCAAAGATATCATAGATCTTTCCGACACCGATCACATC
TTTTCCTTCTGCTTTTAATGCATCCAACACCGTCTGTCTCGGCGGTGCAAGTGAAAAGTCATGCCGT
CTGATTGTCCGTTCGTAATTTGGCCATGTTCCGATGAATGGTCTTGCGATCACTCTTCCGACTCCGA
GATCTCCAGTCAGCATCTCTCTGGCAATCTTACAATATT. Further, assay information is given in previous publications.[1,2] The 50 validated qPCR assays can be ordered from ThermoFisher Scientific (catalogue number 4471120 Custom OpenArray and referring to specific Custom OpenArray ID name: BIOMEPMP-001 ver 1).

Supplementary Note 3. Comparison of the quantitative PCR analyses and metagenome sequencing

Our major finding was that *C. comes*, *D. longicatena*, and *E. ventriosum* associated with ALM. To further validate the assays for these bacteria, we compared the quantitative PCR method with gold standard shotgun metagenome sequencing. The shotgun sequencing library was prepared using MGIEasy FS DNA Library Prep Set (MGI Tech Co., Ltd, China) following the manufacturer's instructions. The sample sequencing libraries together with PCR controls were pooled and sequenced using DNBSEQ-G400 (MGI Tech Co., Ltd) producing at least 20M reads (PE150) per sample. The sequencing reads were processed using StaG-mwc workflow[3] (v0.4.0), which conducts reads quality trimming and filtering using fastp[4] (version 0.20.0), host reads removal using Kraken 2[5] (version 2.0.8_beta), and bacterial taxonomic profiling using MetaPhlAn2[6] (version 2.9.21). Default parameters from the workflow were used for reads processing and taxonomic classification. The relative abundance value for the metagenomic sequencing data was calculated for the 50 species used in the qPCR assay. When evaluated in 130 samples, the quantitative PCR analysis and metagenome sequencing showed high correlation for these three bacteria (*C. comes:* $r=0.99$, $P=3.2\times10^{-115}$; *D. longicatena:* $r=0.77$, $P=4.9\times10^{-27}$; *E.ventriosum:* $r=0.95$, $P=2.1\times10^{-64}$).

Supplementary Note 4. Genotyping

DNA was obtained from blood and genotyping was performed using one of three Illumina HumanCoreExome arrays (Illumina, Inc., San Diego, CA, USA): 12 v.1.0, 12 v.1.1, and 24 with custom content (UM HUNT

Biobank v1.0) at the NTNU Genomic Core Facility (Trondheim, Norway).[7] Samples were excluded if they had contamination > 2.5% as estimated with BAF Regress,[8] large chromosomal copy number variants, lower call rate of a technical duplicate pair and twins, or gonosomal constellations other than XX and XY. Samples were also excluded if they failed to reach a 99% call rate or whose inferred sex contradicted the reported gender. Samples that passed quality control were analyzed in a second round of genotype calling following the Genome Studio quality control protocol.[9] Genomic position, strand orientation, and the reference allele of genotyped variants were determined by aligning their probe sequences against the human genome (Genome Reference Consortium Human genome (build 37) and revised Cambridge Reference Sequence of the human mitochondrial DNA (GenBank ID NC_012920); http://genome.ucsc.edu, accessed Feb 9th 2022) using BLAT (build 2016).[10] Variants were excluded if (1) their probe sequences could not be perfectly mapped to the reference genome, cluster separation was < 0.3, Gentrain score was < 0.15, showed deviations from Hardy Weinberg equilibrium in unrelated samples of European ancestry with p-value < 0.0001), their call rate was < 99%, or another assay with higher call rate genotyped the same variant. The genotyping resulted in approximately 600,000 genetic variants; following imputation (HRC V1.1), approximately 25 million gene variants were obtained.

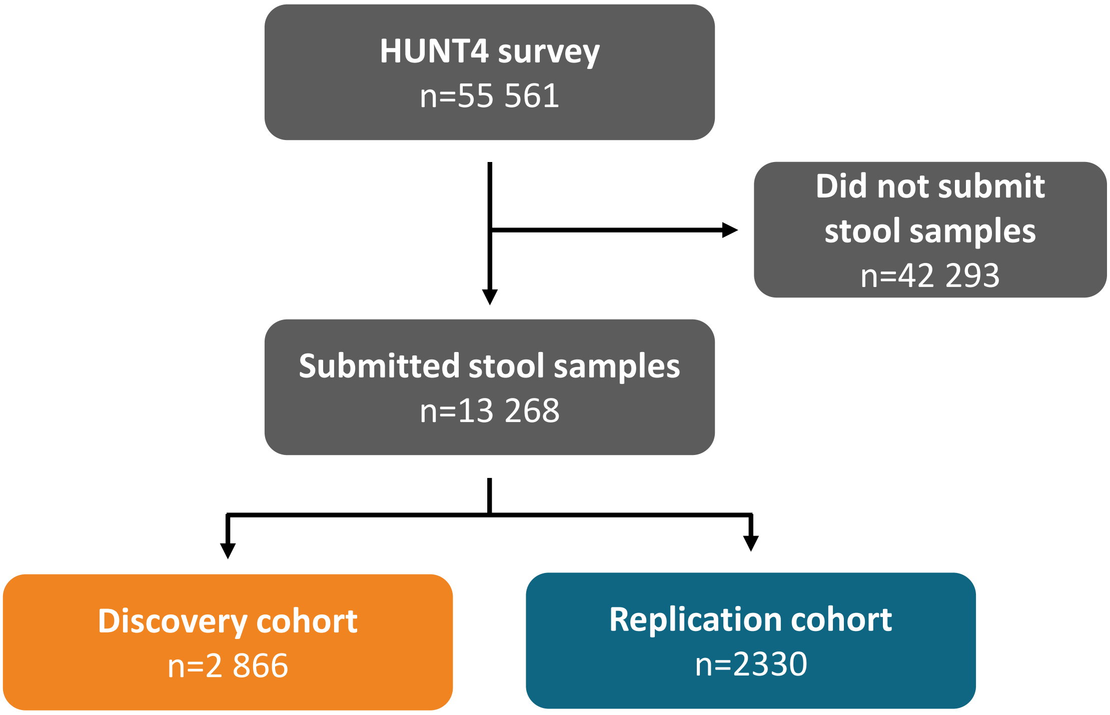

Supplementary Figure 1. Flow chart.

Supplementary Table 1. Study characteristics

	Discovery		Replication		Combined	
	Women (n=1703)	Men (n=1163)	Women (n=1614)	Men (n=716)	Women (n=3317)	Men (n=1879)
**Continous variable**	Mean (SD)	Mean (SD)	Mean (SD)	Mean (SD)	Mean (SD)	Mean (SD)
Age (years)	58.6 (14.1)	62.9 (13.1)	53.4 (13.9)	54.7 (14.3)	56.1 (14.2)	59.7 (14.2)
Height (cm)	164.9 (6.2)	177.6 (6.6)	166.0 (6.0)	178.5 (6.9)	165.4 (6.1)	178 (6.7)
Weight (kg)	74.2 (14.4)	87.3 (13.9)	72.1 (13.0)	85.9 (13.9)	73.1 (13.8)	86.8 (13.9)
BMI (kg/m^2)	27.3 (4.9)	27.7 (4.0)	26.2 (4.5)	26.9 (4.1)	26.7 (4.8)	27.4 (4.0)
Appendicular lean mass (kg)	19.0 (2.7)	26.6 (3.5)	19.2 (2.4)	27.3 (3.5)	19.1 (2.6)	26.9 (3.5)
Total lean mass (kg)	43.7 (5.6)	59.8 (7.3)	44.0 (5.1)	61.0 (7.4)	43.8 (5.4)	60.3 (7.4)
Femoral neck BMD (g/cm^2)	0.78 (0.12)[h]	0.88 (0.14)[h]	0.80 (0.12)[h]	0.89 (0.14)[g]	0.79 (0.12)[j]	0.88 (0.14)[i]
Total hip BMD (g/cm^2)	0.85 (0.13)[h]	0.98 (0.15)[h]	0.87 (0.13)[h]	0.98 (0.14)[g]	0.86 (0.13)[j]	0.88 (0.14)[i]
Coprococcus comes relative abundance (%)	0.7 (0.3-1.2)	0.7 (0.3-1.3)	0.7 (0.3-1.1)	0.7 (0.3-1.2)	0.7 (0.3-1.2)	0.7 (0.3-1.3)
Dorea longicatena relative abundance (%)	1.0 (0.5-1.8)	1.1 (0.5-1.9)	1.1 (0.6-1.8)	1.1 (0.6-1.8)	1.1 (0.5-1.8)	1.1 (0.6-1.9)
Eubacterium ventriosum relative abundance (%)	0.1 (0.0-0.6)	0.1 (0.0-0.6)	0.2 (0.0-0.6)	0.2 (0.0-0.6)	0.1 (0.0-0.6)	0.2 (0.0-0.6)
**Categorical variables**	n (%)	n (%)	n (%)	n (%)	n (%)	n (%)
Coprococcus comes presence	1512 (88.8)	1061 (91.2)	1465 (90.8)	667 (93.2)	2977 (89.8)	1728 (92.0)
Dorea longicatena presence	1547 (90.8)	1070 (92.0)	1484 (91.9)	681 (95.1)	3031 (91.4)	1751 (93.2)
Eubacterium ventriosum presence	942 (55.3)	684 (58.8)	980 (60.7)	488 (68.2)	1922 (57.9)	1172 (62.4)
Involuntary weight loss	67 (4.1)[e]	43 (3.9)[d]	46 (2.9)[d]	28 (4.0)[b]	113 (3.5)[f]	71 (3.9)[e]
Living in Norway at 1 year of age	1638 (96.2)[a]	1134 (97.6)[a]	1547 (95.8)	684 (95.5)	3185 (96)[a]	1818 (96.8)[a]
Chronic disease	822 (48.9)[c]	531 (46.5)[c]	573 (36)[c]	205 (28.8)[a]	1395 (42.6)[d]	736 (39.7)[c]
Use of prescription medication	1225 (72.3)[a]	846 (73.1)[a]	770 (48.0)[a]	195 (27.3)[a]	1995 (60.5)[b]	1041 (55.7)[a]
Current smoker	160 (9.4)[a]	65 (5.6)[a]	138 (8.6)[a]	43 (6.0)[a]	298 (9.0)[b]	108 (5.8)[b]
Stool consistency	[f]	[e]	[e]	[d]	[f]	[f]
Hard	351 (22.0)	156 (14.5)	303 (19.9)	87 (12.9)	654 (21.0)	243 (13.9)
Intermediate	1072 (67.1)	787 (73.1)	1041 (68.4)	493 (73.4)	2113 (67.7)	1280 (73.2)
Loose	174 (10.9)	134 (12.4)	178 (11.7)	92 (13.7)	352 (11.3)	226 (12.9)
Fruit/berries intake frequency	[b]	[b]	[b]	[a]	[c]	[b]
<1 time/week	76 (4.5)	96 (8.3)	61 (3.8)	56 (7.9)	137 (4.2)	152 (8.2)
1-3 times/week	472 (27.9)	406 (35.3)	449 (28.1)	286 (40.2)	921 (28.0)	692 (37.1)
4-6 times/week	615 (36.4)	402 (34.9)	620 (38.8)	236 (33.1)	1235 (37.5)	638 (34.2)
7 or more times/week	526 (31.1)	247 (21.5)	470 (29.4)	134 (18.8)	996 (30.3)	381 (20.5)
Vegetables intake frequency	[b]	[b]	[b]	[a]	[c]	[b]
<1 time/week	21 (1.2)	29 (2.5)	14 (0.9)	15 (2.1)	35 (1.1)	44 (2.4)
1-3 times/week	274 (16.2)	290 (25.3)	284 (17.7)	198 (27.7)	558 (17.0)	488 (26.2)
4-6 times/week	828 (49)	606 (52.8)	779 (48.7)	369 (51.6)	1607 (48.8)	975 (52.3)
7 or more times/week	568 (33.6)	223 (19.4)	524 (32.7)	133 (18.6)	1092 (33.2)	356 (19.1)
Red meat intake frequency	[c]	[b]	[b]	[a]	[d]	[c]
<1 time/week	445 (26.5)	218 (19.1)	398 (24.8)	127 (17.9)	843 (25.7)	345 (18.6)
1-3 times/week	1088 (64.9)	819 (71.6)	1054 (65.8)	503 (70.9)	2142 (65.3)	1322 (71.3)
4-6 times/week	143 (8.5)	98 (8.6)	142 (8.9)	72 (10.2)	285 (8.7)	170 (9.2)
7 or more times/week	1 (0.1)	9 (0.8)	8 (0.5)	7 (1.0)	9 (0.3)	16 (0.9)
White meat intake frequency	[c]	[b]	[b]	[a]	[d]	[c]
<1 time/week	695 (41.4)	533 (46.6)	574 (36)	269 (38)	1269 (38.8)	802 (43.3)
1-3 times/week	909 (54.2)	579 (50.6)	951 (59.6)	424 (60)	1860 (56.8)	1003 (54.2)
4-6 times/week	70 (4.2)	29 (2.5)	66 (4.1)	14 (2)	136 (4.2)	43 (2.3)
7 or more times/week	4 (0.2)	3 (0.3)	4 (0.3)	0 (0.0)	8 (0.2)	3 (0.2)
Processed meat intake frequency	[c]	[b]	[c]	[a]	[d]	[b]
<1 time/week	530 (31.6)	330 (28.7)	442 (27.8)	164 (23.1)	972 (29.7)	494 (26.6)
1-3 times/week	1074 (64.0)	763 (66.4)	1081 (68)	502 (70.7)	2155 (65.9)	1265 (68)
4-6 times/week	74 (4.4)	51 (4.4)	66 (4.2)	42 (5.9)	140 (4.3)	93 (5)
7 or more times/week	0 (0.0)	5 (0.4)	1 (0.1)	2 (0.3)	1 (0.0)	7 (0.4)
Low-fat fish intake frequency	[b]	[a]	[b]	[a]	[c]	[a]
<1 time/week	529 (31.3)	309 (26.8)	576 (36.0)	250 (35)	1105 (33.6)	559 (29.9)
1-3 times/week	1071 (63.4)	777 (67.3)	956 (59.7)	441 (61.8)	2027 (61.6)	1218 (65.2)
4-6 times/week	87 (5.1)	63 (5.5)	66 (4.1)	23 (3.2)	153 (4.6)	86 (4.6)
7 or more times/week	3 (0.2)	6 (0.5)	4 (0.2)	0 (0.0)	7 (0.2)	6 (0.3)
High-fat fish intake frequency	[b]	[a]	[a]	[a]	[c]	[b]
<1 time/week	492 (29.1)	360 (31.2)	483 (30.1)	248 (34.7)	975 (29.6)	608 (32.6)
1-3 times/week	1057 (62.5)	690 (59.8)	992 (61.8)	422 (59.1)	2049 (62.2)	1112 (59.6)
4-6 times/week	121 (7.2)	79 (6.9)	118 (7.4)	34 (4.8)	239 (7.3)	113 (6.1)
7 or more times/week	20 (1.2)	24 (2.1)	11 (0.7)	10 (1.4)	31 (0.9)	34 (1.8)
Intake freuqency of ≤6 glasses of alcohol	[d]	[c]	[d]	[a]	[e]	[d]
Never	1132 (68.4)	563 (49.6)	971 (61.4)	284 (39.9)	2103 (65.0)	847 (45.9)
Less than monthly	446 (27)	425 (37.4)	518 (32.7)	308 (43.3)	964 (29.8)	733 (39.7)
Monthly	67 (4.1)	119 (10.5)	82 (5.2)	97 (13.6)	149 (4.6)	216 (11.7)
Weekly	7 (0.4)	25 (2.2)	10 (0.6)	22 (3.1)	17 (0.5)	47 (2.5)
Daily	2 (0.1)	3 (0.3)	1 (0.1)	0 (0.0)	3 (0.1)	3 (0.2)

Continous parameters are given as mean (standard deviation, SD) except for the relative abundance of bacteria where data are median (interquartile range). Categorical variables are given as number of events (%). Missing n: [a]≤ 10, [b]11-20, [c]21-28, [d]32-55, [e]65-92, [f]104-198, [g]414, [h]754-999, [i]1168, [j]1878-1883

Supplementary Table 2. Associations between microbes and appendicular mass in the discovery cohort

									Microbioal abundance (binary) vs appendicular lean mass (n=2866)					Microbioal abundance (quartile) vs appendicular lean mass (n=2866)				
Kingdom	Domain	Phylum	Class	Order	Family	Genus	Species	% detectable	β	SE	LCI	UCI	P	β	SE	LCI	UCI	P
Bacteria	*Bacteria*	*Actinobacteria*	*Actinobacteria*	*Bifidobacteriales*	*Bifidobacteriaceae*	*Bifidobacterium*	*Bifidobacterium adolescentis*	52.7	0.09	0.07	-0.05	0.23	0.211	0.03	0.03	-0.03	0.09	0.266
Bacteria	*Bacteria*	*Actinobacteria*	*Actinobacteria*	*Bifidobacteriales*	*Bifidobacteriaceae*	*Bifidobacterium*	*Bifidobacterium angulatum*	2.5			< 10% frequency					< 10% frequency		
Bacteria	*Bacteria*	*Actinobacteria*	*Actinobacteria*	*Bifidobacteriales*	*Bifidobacteriaceae*	*Bifidobacterium*	*Bifidobacterium bifidum*	23.7	0.01	0.08	-0.15	0.18	0.867	-0.03	0.04	-0.10	0.05	0.465
Bacteria	*Bacteria*	*Actinobacteria*	*Actinobacteria*	*Bifidobacteriales*	*Bifidobacteriaceae*	*Bifidobacterium*	*Bifidobacterium catenulatum*	10.2	0.11	0.12	-0.12	0.34	0.351	0.03	0.05	-0.07	0.14	0.515
Bacteria	*Bacteria*	*Actinobacteria*	*Actinobacteria*	*Bifidobacteriales*	*Bifidobacteriaceae*	*Bifidobacterium*	*Bifidobacterium longum*	82.3	0.07	0.09	-0.11	0.25	0.469	-0.04	0.03	-0.11	0.02	0.189
Bacteria	*Bacteria*	*Actinobacteria*	*Actinobacteria*	*Bifidobacteriales*	*Bifidobacteriaceae*	*Bifidobacterium*	*Bifidobacterium pseudocatenulatum*	17.0	-0.10	0.09	-0.28	0.09	0.296	-0.06	0.04	-0.14	0.02	0.155
Bacteria	*Bacteria*	*Actinobacteria*	*Actinobacteria*	*Coriobacteriales*	*Coriobacteriaceae*	*Collinsella*	*Collinsella aerofaciens*	81.6	0.27	0.09	0.09	0.45	0.003	0.06	0.03	-0.01	0.12	0.093
Bacteria	*Bacteria*	*Bacteroidetes*	*Bacteroidia*	*Bacteroidales*	*Bacteroidaceae*	*Bacteroides*	*Bacteroides caccae*	71.4	0.17	0.08	0.02	0.32	0.031	-0.01	0.03	-0.07	0.05	0.835
Bacteria	*Bacteria*	*Bacteroidetes*	*Bacteroidia*	*Bacteroidales*	*Bacteroidaceae*	*Bacteroides*	*Bacteroides cellulosilyticus*	48.4	0.15	0.07	0.01	0.29	0.031	0.06	0.03	0.00	0.12	0.061
Bacteria	*Bacteria*	*Bacteroidetes*	*Bacteroidia*	*Bacteroidales*	*Bacteroidaceae*	*Bacteroides*	*Bacteroides eggerthii*	22.5	0.08	0.08	-0.08	0.25	0.326	0.05	0.04	-0.03	0.12	0.194
Bacteria	*Bacteria*	*Bacteroidetes*	*Bacteroidia*	*Bacteroidales*	*Bacteroidaceae*	*Bacteroides*	*Bacteroides finegoldii*	14.6	0.03	0.10	-0.17	0.23	0.772	0.02	0.05	-0.07	0.11	0.631
Bacteria	*Bacteria*	*Bacteroidetes*	*Bacteroidia*	*Bacteroidales*	*Bacteroidaceae*	*Bacteroides*	*Bacteroides ovatus*	77.9	0.10	0.09	-0.07	0.27	0.242	0.01	0.03	-0.06	0.07	0.831
Bacteria	*Bacteria*	*Bacteroidetes*	*Bacteroidia*	*Bacteroidales*	*Bacteroidaceae*	*Bacteroides*	*Bacteroides stercorirosoris*	1.0			< 10% frequency					< 10% frequency		
Bacteria	*Bacteria*	*Bacteroidetes*	*Bacteroidia*	*Bacteroidales*	*Bacteroidaceae*	*Bacteroides*	*Bacteroides thetaiotaomicron*	76.0	0.16	0.08	0.00	0.33	0.047	-0.03	0.03	-0.09	0.04	0.403
Bacteria	*Bacteria*	*Bacteroidetes*	*Bacteroidia*	*Bacteroidales*	*Bacteroidaceae*	*Bacteroides*	*Bacteroides xylanisolvens*	67.3	0.19	0.08	0.05	0.34	0.010	0.01	0.03	-0.05	0.07	0.854
Bacteria	*Bacteria*	*Bacteroidetes*	*Bacteroidia*	*Bacteroidales*	*Bacteroidaceae*	*Phocaeicola*	*Phocaeicola dorei*	73.2	0.18	0.08	0.02	0.34	0.025	-0.01	0.03	-0.07	0.05	0.839
Bacteria	*Bacteria*	*Bacteroidetes*	*Bacteroidia*	*Bacteroidales*	*Bacteroidaceae*	*Phocaeicola*	*Phocaeicola massiliensis*	28.3	0.19	0.08	0.04	0.35	0.014	0.09	0.04	0.02	0.16	0.013
Bacteria	*Bacteria*	*Bacteroidetes*	*Bacteroidia*	*Bacteroidales*	*Bacteroidaceae*	*Phocaeicola*	*Phocaeicola vulgatus*	86.4	0.06	0.10	-0.15	0.26	0.583	0.05	0.03	-0.02	0.11	0.185
Bacteria	*Bacteria*	*Bacteroidetes*	*Bacteroidia*	*Bacteroidales*	*Porphyromonadaceae*	*Barnesiella*	*Barnesiella intestinihominis*	67.4	0.11	0.08	-0.04	0.25	0.159	0.00	0.03	-0.06	0.06	0.983
Bacteria	*Bacteria*	*Bacteroidetes*	*Bacteroidia*	*Bacteroidales*	*Porphyromonadaceae*	*Parabacteroides*	*Parabacteroides distasonis*	88.2	0.16	0.11	-0.06	0.37	0.152	-0.04	0.04	-0.11	0.03	0.279
Bacteria	*Bacteria*	*Bacteroidetes*	*Bacteroidia*	*Bacteroidales*	*Porphyromonadaceae*	*Parabacteroides*	*Parabacteroides merdae*	70.7	0.20	0.08	0.04	0.35	0.012	0.05	0.03	-0.01	0.11	0.132
Bacteria	*Bacteria*	*Bacteroidetes*	*Bacteroidia*	*Bacteroidales*	*Prevotellaceae*	*Prevotella*	*Prevotella copri*	48.7	0.18	0.07	0.04	0.31	0.013	0.08	0.03	0.02	0.14	0.012
Bacteria	*Bacteria*	*Bacteroidetes*	*Bacteroidia*	*Bacteroidales*	*Rikenellaceae*	*Alistipes*	*Alistipes finegoldii*	12.2	0.18	0.11	-0.03	0.40	0.090	0.05	0.05	-0.05	0.15	0.310
Bacteria	*Bacteria*	*Bacteroidetes*	*Bacteroidia*	*Bacteroidales*	*Rikenellaceae*	*Alistipes*	*Alistipes putredinis*	80.1	0.17	0.09	0.00	0.34	0.055	0.03	0.03	-0.03	0.10	0.297
Bacteria	*Bacteria*	*Firmicutes*	*Bacilli*	*Lactobacillales*	*Lactobacillaceae*	*Lactobacillus*	*Lactobacillus acidophilus*	6.7			< 10% frequency					< 10% frequency		
Bacteria	*Bacteria*	*Firmicutes*	*Bacilli*	*Lactobacillales*	*Lactobacillaceae*	*Lactobacillus*	*Lactobacillus paracasei*	8.0			< 10% frequency					< 10% frequency		
Bacteria	*Bacteria*	*Firmicutes*	*Bacilli*	*Lactobacillales*	*Lactobacillaceae*	*Ligilactobacillus*	*Ligilactobacillus ruminis*	4.7			< 10% frequency					< 10% frequency		
Bacteria	*Bacteria*	*Firmicutes*	*Bacilli*	*Lactobacillales*	*Streptococcaceae*	*Streptococcus*	*Streptococcus thermophilus*	48.3	0.15	0.07	0.01	0.28	0.040	0.05	0.03	-0.01	0.11	0.097
Bacteria	*Bacteria*	*Firmicutes*	*Clostridia*	*Clostridiales*	*Christensenellaceae*	*Christensenella*	*Christensenella minuta*	11.3	-0.25	0.11	-0.47	-0.03	0.024	-0.10	0.05	-0.19	-0.01	0.027
Bacteria	*Bacteria*	*Firmicutes*	*Clostridia*	*Clostridiales*	*Clostridiaceae*	*Clostridium*	*Clostridium leptum*	80.6	-0.22	0.09	-0.39	-0.04	0.014	-0.07	0.03	-0.13	-0.01	0.033
Bacteria	*Bacteria*	*Firmicutes*	*Clostridia*	*Clostridiales*	*Clostridiaceae*	*Clostridium*	*Clostridium scindens*	14.5	-0.31	0.10	-0.51	-0.12	0.002	-0.13	0.04	-0.22	-0.04	0.003
Bacteria	*Bacteria*	*Firmicutes*	*Clostridia*	*Clostridiales*	*Eubacteriaceae*	*Eubacterium*	*Eubacterium ventriosum*	56.7	0.36	0.07	0.22	0.50	**5.2E-07**	0.11	0.03	0.06	0.17	**1.5E-04**
Bacteria	*Bacteria*	*Firmicutes*	*Clostridia*	*Clostridiales*	*Lachnospiraceae*	*Anaerobutyricum*	*Anaerobutyricum hallii*	38.8	0.09	0.07	-0.06	0.23	0.235	0.03	0.03	-0.03	0.09	0.362
Bacteria	*Bacteria*	*Firmicutes*	*Clostridia*	*Clostridiales*	*Lachnospiraceae*	*Blautia*	*Blautia hydrogenotrophica*	13.0	-0.22	0.11	-0.43	-0.02	0.035	-0.13	0.05	-0.22	-0.03	0.008
Bacteria	*Bacteria*	*Firmicutes*	*Clostridia*	*Clostridiales*	*Lachnospiraceae*	*Coprococcus*	*Coprococcus comes*	89.8	0.57	0.12	0.34	0.80	**1.1E-06**	0.17	0.04	0.10	0.24	**1.5E-06**
Bacteria	*Bacteria*	*Firmicutes*	*Clostridia*	*Clostridiales*	*Lachnospiraceae*	*Dorea*	*Dorea formicigenerans*	83.8	0.24	0.10	0.05	0.43	0.012	0.06	0.03	0.00	0.13	0.062
Bacteria	*Bacteria*	*Firmicutes*	*Clostridia*	*Clostridiales*	*Lachnospiraceae*	*Dorea*	*Dorea longicatena*	91.3	0.46	0.13	0.22	0.71	**2.1E-04**	0.12	0.04	0.05	0.19	**1.1E-03**
Bacteria	*Bacteria*	*Firmicutes*	*Clostridia*	*Clostridiales*	*Lachnospiraceae*	*Mediterraneibacter*	*Ruminococcus gnavus*	34.9	-0.28	0.07	-0.42	-0.13	**1.9E-04**	-0.13	0.03	-0.20	-0.07	**4.5E-05**
Bacteria	*Bacteria*	*Firmicutes*	*Clostridia*	*Clostridiales*	*Lachnospiraceae*	*Roseburia*	*Roseburia hominis*	85.9	-0.05	0.10	-0.25	0.15	0.613	0.00	0.03	-0.07	0.07	0.978
Bacteria	*Bacteria*	*Firmicutes*	*Clostridia*	*Clostridiales*	*Lachnospiraceae*	*Roseburia*	*Roseburia intestinalis*	86.5	0.17	0.10	-0.03	0.38	0.095	0.03	0.03	-0.04	0.10	0.418
Bacteria	*Bacteria*	*Firmicutes*	*Clostridia*	*Clostridiales*	*Ruminococcaceae*	*Faecalibacterium*	*Faecalibacterium prausnitzii*	83.8	0.27	0.10	0.08	0.46	0.005	0.05	0.03	-0.02	0.11	0.179
Bacteria	*Bacteria*	*Firmicutes*	*Clostridia*	*Clostridiales*	*Ruminococcaceae*	*Subdoligranulum*	*Subdoligranulum variabile*	0.4			< 10% frequency					< 10% frequency		
Bacteria	*Bacteria*	*Firmicutes*	*Clostridia*	*Eubacteriales*	*Clostridiaceae*	*Clostridium*	*Clostridium ramosum*	15.7	-0.35	0.10	-0.54	-0.16	**2.8E-04**	-0.14	0.04	-0.22	-0.05	0.002
Bacteria	*Bacteria*	*Firmicutes*	*Clostridia*	*Eubacteriales*	*Lachnospiraceae*	*Agathobacter*	*Agathobacter rectalis*	97.0	0.26	0.21	-0.15	0.67	0.216	0.02	0.04	-0.06	0.10	0.574
Bacteria	*Bacteria*	*Firmicutes*	*Clostridia*	*Eubacteriales*	*Lachnospiraceae*	*Lachnospira*	*Lachnospira eligens*	81.0	0.14	0.09	-0.04	0.31	0.135	-0.01	0.03	-0.07	0.05	0.754
Bacteria	*Bacteria*	*Proteobacteria*	*Beta Proteobacteria*	*Burkholderiales*	*Sutterellaceae*	*Sutterella*	*Sutterella wadsworthensis*	46.4	0.17	0.07	0.03	0.31	0.017	0.04	0.03	-0.02	0.10	0.163
Bacteria	*Bacteria*	*Proteobacteria*	*Gammaproteobacteria*	*Enterobacteriales*	*Enterobacteriaceae*	*Escherichia*	*Escherichia coli*	57.1	-0.03	0.07	-0.17	0.11	0.707	-0.04	0.03	-0.10	0.02	0.174
Bacteria	*Bacteria*	*Proteobacteria*	*Gammaproteobacteria*	*Enterobacteriales*	*Enterobacteriaceae*	*Klebsiella*	*Klebsiella variicola*	2.6			< 10% frequency					< 10% frequency		
Bacteria	*Bacteria*	*Verrucomicrobia*	*Verrucomicrobiae*	*Verrucomicrobiales*	*Akkermansiaceae*	*Akkermansia*	*Akkermansia muciniphila*	74.0	-0.04	0.08	-0.20	0.12	0.645	-0.05	0.03	-0.11	0.01	0.098
Euryarchaeota	*Archaea*	*Euryarchaeota*	*Methanobacteria*	*Methanobacteriales*	*Methanobacteriaceae*	*Methanobrevibacter*	*Methanobrevibacter smithii*	46.9	-0.09	0.07	-0.23	0.05	0.194	-0.09	0.03	-0.15	-0.02	0.006

Linear regressions (for microbes with >10% frequency in the discovery cohort) including bacterial relative abundance as exposure and appendicular lean mass as outcome, adjusted for age, gender, height, and body fat mass. The tests were 2-sided. The relative abundance data were divided into 2 (binary) or 4 quantiles (quartiles), with all the non-detectable values in the first quantile. The data from the linear regression are presented as the estimated associations (β values in kg/microbial quartile or binary step with standard error of mean [SE] and 95% confidence intervalls [CIs]). Undetectable values are represented as 0. Bonferroni correction was used to adjust for multiple comparisons and 0.0012 (0.05/43) was considered signficant; significant results are in shown in bold. SE, standard error; LCI, lower 95% confidence interval; UCI, upper 95% confidence interval

Supplementary Table 3. Associations between microbial species and appendicular lean mass without adjustment for body fat mass

	Microbial abundance (binary)					Microbial abundance (quartiles)				
	β	SE	LCI	UCI	P	β	SE	LCI	UCI	P
Clostridium ramosum	-0.15	0.11	-0.37	0.06	0.16	-0.06	0.05	-0.16	0.04	0.23
Coprococcus comes	0.47	0.13	0.21	0.73	4.3E-04	0.14	0.04	0.06	0.22	4.3E-04
Dorea longicatena	0.40	0.14	0.13	0.68	4.6E-03	0.09	0.04	0.01	0.17	0.028
Eubacterium ventriosum	0.34	0.08	0.18	0.50	3.3E-05	0.08	0.03	0.01	0.15	0.017
Ruminococcus gnavus	-0.05	0.08	-0.21	0.12	0.59	-0.02	0.04	-0.10	0.05	0.54

Microbial species significantly associated (passing Bonferroni correction) with appendicular lean mass using the basic model (adjusted for gender, age, height, and body fat mass) were further analyzed using the basic model without body fat mass adjustment. The tests were 2-sided. The relative abundance data were divided into 2 (binary) or 4 (quartile) quantiles, with all the non-detectable values in the first quantile, and analyzed using linear regression. The β values are given in kg/microbial quartile or binary step. Bacterial species in bold were significantly associated with appendicular lean mass and thus fullfilled the criteria for being eligble for replication. n=2866. SE, standard error; LCI, lower 95% confidence interval; UCI, upper 95% confidence interval

Supplementary Table 4. Replication of significant associations between the presence of microbial species and appendicular lean mass using binary data

	Discovery (n=2866)					Replication (n=2330)					Combined (n=5196)				
	β	SE	LCI	UCI	P	β	SE	LCI	UCI	P	β	SE	LCI	UCI	P
Coprococcus comes	0.57	0.12	0.34	0.80	1.1E-06	0.35	0.14	0.08	0.62	1.1E-02	0.48	0.09	0.30	0.65	7.9E-08
Dorea longicatena	0.46	0.13	0.22	0.71	2.1E-04	0.40	0.15	0.11	0.69	7.3E-03	0.45	0.10	0.26	0.64	3.1E-06
Eubacterium ventriosum	0.36	0.07	0.22	0.50	5.2E-07	0.17	0.08	0.01	0.33	3.2E-02	0.28	0.05	0.18	0.39	1.1E-07

Replication of associations in the discovery cohort using linear regressions with relative abundance of microbial species (as binary data, i.e. presence/absence) as exposure and appendicular lean mass as the outcome, adjusted for age, gender, height, and body fat mass (and cohort- and batch-specific covariates when needed, i.e. basic adjustment) for the discovery, replication, and combined cohorts. The tests were 2-sided. The β values are given in kg for presence of microbes. SE, standard error; LCI, lower 95% confidence interval; UCI, upper 95% confidence interval

Supplementary Table 5. Replication of significant associations between relative abundance of microbial species and appendicular lean mass using quartile data

	Discovery (n=2866)					Replication (n=2330)					Combined (n=5196)				
	β	SE	LCI	UCI	P	β	SE	LCI	UCI	P	β	SE	LCI	UCI	P
Coprococcus comes	0.17	0.04	0.10	0.24	1.5E-06	0.07	0.040	-0.01	0.15	7.8E-02	0.13	0.03	0.08	0.18	1.2E-06
Dorea longicatena	0.12	0.04	0.05	0.19	1.1E-03	0.14	0.040	0.06	0.21	8.5E-04	0.13	0.03	0.08	0.18	2.1E-06
Eubacterium ventriosum	0.11	0.03	0.06	0.17	1.5E-04	0.01	0.033	-0.06	0.07	8.8E-01	0.07	0.02	0.03	0.11	2.0E-03

Replication of associations in the discovery cohort using linear regressions with relative abundance of microbial species (in quartiles) as exposure and appendicular lean mass as the outcome, adjusted for age, gender, height, and body fat mass (and cohort- and batch specific covariates when needed, i.e. basic adjustment) for the discovery, replication, and combined cohorts. The tests were 2-sided. The microbial relative abundance data was divided into quartiles, with all the non-detectable values in the first quartile. The β values are given in kg/microbial quartile. SE, standard error; LCI, lower 95% confidence interval; UCI, upper 95% confidence interval

Supplementary Table 6. Sensitivity analyses of significant associations between relative abundance of microbial species and appendicular lean mass using CLR-transformed data

	Discovery (n=2866)					Replication (n=2330)					Combined (n=5196)				
	**β**	**SE**	**LCI**	**UCI**	**P**	**β**	**SE**	**LCI**	**UCI**	**P**	**β**	**SE**	**LCI**	**UCI**	**P**
Coprococcus comes	0.17	0.03	0.10	0.23	9.4E-07	0.09	0.04	0.01	0.17	2.7E-02	0.14	0.03	0.08	0.19	1.8E-07
Dorea longicatena	0.11	0.03	0.05	0.18	8.5E-04	0.13	0.04	0.05	0.21	1.2E-03	0.12	0.03	0.07	0.17	2.4E-06
Eubacterium ventriosum	0.15	0.03	0.08	0.22	1.7E-05	0.02	0.04	-0.05	0.10	5.7E-01	0.10	0.03	0.05	0.15	1.8E-04

Sensitivity analyses of associations in the discovery and replication cohorts using linear regressions with CLR-transformed relative abundance of microbial species as exposure and appendicular lean mass as the outcome, adjusted for age, gender, height, and body fat mass (and cohort- and batch-specific covariates when needed, i.e. basic adjustment) for the discovery, replication, and combined cohorts. The tests were 2-sided. The β values are given in kg/standard deviation CLR-transformed relative abundance. SE, standard error; LCI, lower 95% confidence interval; UCI, upper 95% confidence interval

Supplementary Table 7. Associations for the presence of *Coprococcus comes*, *Dorea longicatena,* and *Eubacterium ventriosum* with appendicular lean mass and total lean mass after different adjustments

	Appendicular mass					Total lean mass					
	β	SE	LCI	UCI	P	β	SE	LCI	UCI	P	N
Coprococcus comes											
Basic model	0.48	0.09	0.30	0.65	7.9E-08	0.97	0.18	0.61	1.34	1.3E-07	5196
Intermediate model	0.45	0.09	0.27	0.63	1.6E-06	0.93	0.19	0.55	1.31	1.9E-06	4773
Full model	0.43	0.10	0.24	0.61	1.0E-05	0.88	0.20	0.49	1.27	1.1E-05	4535
Dorea longicatena											
Basic model	0.45	0.10	0.26	0.64	3.1E-06	0.91	0.20	0.52	1.30	4.9E-06	5196
Intermediate model	0.42	0.10	0.22	0.62	3.3E-05	0.86	0.21	0.45	1.27	4.4E-05	4773
Full model	0.38	0.10	0.18	0.59	2.1E-04	0.78	0.21	0.36	1.20	2.9E-04	4535
Eubacterium ventriosum											
Basic model	0.28	0.05	0.18	0.39	1.1E-07	0.57	0.11	0.35	0.78	2.8E-07	5196
Intermediate model	0.25	0.06	0.14	0.36	4.7E-06	0.52	0.11	0.29	0.74	7.0E-06	4773
Full model	0.22	0.06	0.11	0.33	8.0E-05	0.46	0.12	0.23	0.69	9.9E-05	4535

The linear regressions for the combined cohort (discovery and replication cohorts) included presence or absence of *Dorea longicatena, Coprococcus comes,* or *Eubacterium ventriosum* as exposure and appendicular lean mass or total lean mass as the outcome. The tests were 2-sided. Adjustments were: the basic model – age, gender, height, body fat mass, and cohort- and batch-specific covariates; the intermediate model – basic model plus chronic disease, smoking status, stool consistency, and use of prescription medication; and the full model – intermediate model plus alcohol and food intake frequency. The β values are given in kg for presence of microbes. SE, standard error; LCI, lower 95% confidence interval; UCI, upper 95% confidence interval

Supplementary Table 8. Stratified associations for the presence of *Coprococcus comes, Dorea longicatena,* and *Eubacterium ventriosum* with appendicular lean mass and total lean mass

	Appendicular lean mass					Total lean mass					
	β	SE	LCI	UCI	P	β	SE	LCI	UCI	P	N
Coprococcus comes											
All	0.168	0.030	0.110	0.226	1.7E-08	0.164	0.029	0.106	0.221	2.3E-08	5196
Women	0.173	0.035	0.104	0.242	9.5E-07	0.171	0.035	0.103	0.239	8.8E-07	3317
Men	0.139	0.054	0.033	0.246	1.0E-02	0.135	0.054	0.030	0.240	1.2E-02	1879
Young	0.165	0.043	0.080	0.251	1.4E-04	0.167	0.043	0.084	0.251	8.9E-05	2591
Old	0.164	0.039	0.088	0.240	2.6E-05	0.156	0.039	0.080	0.233	6.1E-05	2605
Dorea longicatena											
All	0.164	0.032	0.101	0.227	3.2E-07	0.159	0.032	0.098	0.221	4.6E-07	5196
Women	0.154	0.038	0.079	0.228	5.8E-05	0.150	0.038	0.076	0.223	6.8E-05	3317
Men	0.163	0.058	0.048	0.277	5.4E-03	0.161	0.058	0.047	0.274	5.5E-03	1879
Young	0.152	0.050	0.054	0.250	2.3E-03	0.152	0.049	0.056	0.248	1.9E-03	2591
Old	0.140	0.040	0.062	0.218	4.3E-04	0.137	0.040	0.059	0.215	5.6E-04	2605
Eubacterium ventriosum											
All	0.090	0.018	0.056	0.125	3.5E-07	0.086	0.017	0.052	0.120	9.4E-07	5196
Women	0.081	0.022	0.039	0.124	1.8E-04	0.076	0.021	0.034	0.118	3.8E-04	3317
Men	0.105	0.030	0.045	0.164	6.0E-04	0.102	0.030	0.043	0.161	7.6E-04	1879
Young	0.102	0.026	0.050	0.154	1.1E-04	0.098	0.026	0.047	0.149	1.6E-04	2591
Old	0.064	0.023	0.019	0.109	5.1E-03	0.062	0.023	0.017	0.107	7.2E-03	2605

Linear regressions with presence or absence of microbial species as exposure and appendicular lean mass and total lean mass as the outcome, adjusted for age, gender, height, body fat mass, and cohort- and batch-specific covariates for the combined cohorts. The tests were 2-sided. The β values are given in standard deviations for the presence of microbes. SE, standard error; LCI, lower 95% confidence interval; UCI, upper 95% confidence interval

Supplementary Table 9. Combined model evaluating the associations between the presence of *Coprococcus comes*, *Dorea longicatena,* and *Eubacterium ventriosum* and lean mass

	Appendicular lean mass					Total lean mass				
	β	SE	LCI	UCI	P	β	SE	LCI	UCI	P
Coprococcus comes	0.33	0.10	0.14	0.52	6.1E-04	0.67	0.20	0.28	1.06	7.0E-04
Dorea longicatena	0.24	0.10	0.04	0.44	2.1E-02	0.49	0.21	0.06	0.91	2.4E-02
Eubacterium ventriosum	0.21	0.05	0.11	0.32	9.4E-05	0.43	0.11	0.20	0.65	1.8E-04

Combined linear regression model including presence or absence of *Dorea longicatena*, *Coprococcus comes*, and *Eubacterium ventriosum* as exposures and appendicular lean mass or total lean mass as the outcomes. The tests were 2-sided. Adjustments – age, gender, height, body fat mass, and cohort- and batch-specific covariates. The b values are given in kg for presence of microbes. n=5196. SE, standard error; LCI, lower 95% confidence interval; UCI, upper 95% confidence interval

Supplementary Table 10. Associations between anabolic species count and lean mass in individuals living in Norway at 1 year of age

	Appendicular lean mass					Total lean mass					
	β	SE	LCI	UCI	P	β	SE	LCI	UCI	P	N
All	0.052	0.007	0.038	0.066	5.6E-13	0.050	0.007	0.036	0.064	2.2E-12	5196
Living in Norway at 1 year of age	0.053	0.007	0.039	0.068	5.7E-13	0.051	0.007	0.036	0.065	2.8E-12	5003

Linear regressions with anabolic species count as exposure and appendicular lean mass and total lean mass as the outcome, adjusted for age, gender, height, body fat mass, and cohort- and batch-specific covariates. The tests were 2-sided. The β values are given in standard deviations per anabolic species count. SE, standard error; LCI, lower 95% confidence interval; UCI, upper 95% confidence interval

Supplementary Table 11. Correlation between anabolic bacterial species and circulating metabolites

Metabolite	Spearman's rho	*p*-value
Coprococcus comes		
Negative associations		
p-cresol sulfate	-0.16	1.72E-46
phenylacetate	-0.14	5.09E-39
p-cresol glucuronide	-0.13	7.32E-35
5-hydroxyhexanoate	-0.13	1.91E-32
Positive associations		
cortolone glucuronide (1)	0.12	4.54E-29
metabolonic lactone sulfate	0.12	8.85E-27
N-stearoyl-sphingosine (d18:1/18:0)	0.11	1.44E-22
4-hydroxyglutamate	0.10	9.14E-21
Dorea longicatena		
Negative associations		
p-cresol sulfate	-0.15	1.07E-42
3b-hydroxy-5-cholenoic acid	-0.13	2.47E-32
p-cresol glucuronide	-0.12	2.72E-28
5-hydroxyhexanoate	-0.12	2.69E-28
Positive associations		
2-piperidinone	0.16	3.55E-50
ursodeoxycholate	0.14	8.41E-36
metabolonic lactone sulfate	0.12	1.21E-29
cortolone glucuronide (1)	0.11	5.99E-24
Eubacterium ventriosum		
Negative associations		
phenylacetylglutamine	-0.10	2.29E-21
p-cresol sulfate	-0.10	1.88E-19
p-cresol glucuronide	-0.10	1.27E-19
phenylacetate	-0.10	1.28E-18
Positive associations		
1,5-anhydroglucitol (1,5-AG)	0.07	7.32E-11
xanthurenate	0.06	5.08E-09
indolepropionate	0.06	4.77E-09
2-piperidinone	0.06	5.15E-08

Table shows top 4 negative and top 4 positive correlations (2-sided) per bacteria from the dataset by Dekkers et al (Nat Commun 2022). Ranking is based on Spearman's rho for negative and positive associations, respectively, among the 1052 annotated metabolites out of the 1321 total metabolites. n=8583

Supplementary Table 12. Descriptives comparing the different subgroups of the HUNT4 study

	HUNT4 (n=55561)	Individuals without feces samples (n=42293)	Individuals with feces samples (n=13268)	Discovery (n=2866)	Replication (n=2330)	Combined (n=5196)	Combined cohort with BMD data (n=1028)
All							
Women (%, n)[a]	55.0 (30534)	53.1 (22440)	61.0 (8094)	59.4 (1703)	69.3 (1614)	63.8 (3317)	71.4 (734)
Age (yr)	54.9 (17.8)	53.5 (18.6)	59.6 (14.2)	60.3 (13.9)	53.8 (14.0)	57.4 (14.3)	60.7 (12.3)
Appendicular lean mass (kg)	22.8 (5.2)[b]	23.1 (5.3)[c]	22.0 (4.8)[d]	22.1 (4.8)	21.7 (4.7)	21.9 (4.8)	21.2 (4.6)
Total lean mass (kg)	51.6 (10.9)[b]	52.2 (11.1)[c]	50.0 (10.2)[d]	50.2 (10.2)	49.2 (9.8)	49.8 (10.0)	48.4 (9.8)
Women							
Age (yr)	54.6 (18.1)	53.2 (19.1)	58.4 (14.4)	58.6 (14.1)	53.4 (13.9)	56.1 (14.2)	60.1 (12.5)
Appendicular lean mass (kg)	19.0 (2.7)[e]	19.1 (2.8)[f]	18.9 (2.5)[g]	19.0 (2.7)	19.2 (2.4)	19.1 (2.6)	18.9 (2.4)
Total lean mass (kg)	43.8 (5.7)[e]	43.8 (5.8)[f]	43.6 (5.4)[g]	43.7 (5.6)	44.0 (5.1)	43.8 (5.4)	43.4 (5.1)
Men							
Age (yr)	55.3 (17.5)	53.7 (18.0)	61.6 (13.6)	62.9 (13.1)	54.7 (14.3)	59.7 (14.2)	62.3 (11.8)
Appendicular lean mass (kg)	27.3 (3.8)[h]	27.4 (3.8)[i]	26.8 (3.5)[j]	26.6 (3.5)	27.3 (3.5)	26.9 (3.5)	27.1 (3.5)
Total lean mass (kg)	61.0 (7.9)[h]	61.3 (8.0)[i]	60.2 (7.4)[j]	59.8 (7.3)	61.0 (7.4)	60.3 (7.4)	60.8 (7.2)

Data are mean (standard deviation), unless otherwise specified.[a]Data are % (n), missing n = [b]4399, [c]3901, [d]498, [e]2673, [f]2375, [g]298, [h]1726, [i]1526, [j]200

SUPPLEMENTARY REFERENCES

1. Farup, P.G. & Maseng, M.G. Are Faecal Microbiota Analyses on Species-Level Suitable Clinical Biomarkers? A Pilot Study in Subjects with Morbid Obesity. *Microorganisms* **9**(2021).
2. Grahnemo, L., *et al.* Cross-sectional associations between the gut microbe Ruminococcus gnavus and features of the metabolic syndrome. *Lancet Diabetes Endo* **10**, 481-483 (2022).
3. Boulund, F. boulund/stag-mwc: StaG-mwc v0.4.0. (Zenodo, 2020).
4. Chen, S., Zhou, Y., Chen, Y. & Gu, J. fastp: an ultra-fast all-in-one FASTQ preprocessor. *Bioinformatics* **34**, i884-i890 (2018).
5. Wood, D.E., Lu, J. & Langmead, B. Improved metagenomic analysis with Kraken 2. *Genome Biol* **20**, 257 (2019).
6. Truong, D.T., *et al.* MetaPhlAn2 for enhanced metagenomic taxonomic profiling. *Nat Methods* **12**, 902-903 (2015).
7. Brumpton, B.M., *et al.* The HUNT study: A population-based cohort for genetic research. *Cell Genomics* **2**, 100193 (2022).
8. Jun, G., *et al.* Detecting and estimating contamination of human DNA samples in sequencing and array-based genotype data. *Am J Hum Genet* **91**, 839-848 (2012).
9. Guo, Y., *et al.* Illumina human exome genotyping array clustering and quality control. *Nat Protoc* **9**, 2643-2662 (2014).
10. Consortium, E.P. An integrated encyclopedia of DNA elements in the human genome. *Nature* **489**, 57-74 (2012).
11. Asvold, B.O., *et al.* Cohort Profile Update: The HUNT Study, Norway. *Int J Epidemiol*, DOI: 10.1093/ije/dyac1095 (2022).
