# Supplementary material for: Identification of three bacterial species associated with increased appendicular lean mass: the HUNT study
Source: Nat Commun. 2023 Apr 20;14:2250. doi: 10.1038/s41467-023-37978-9 (PMC10119287; doi:10.1038/s41467-023-37978-9)
Supplement: Supplementary file 2 — Description of Additional Supplementary Files [file 41467_2023_37978_MOESM2_ESM.pdf]

Description of Additional Supplementary Files

File Name: Supplementary Data 1
Description: Estimated causal effects from the individual genetic variants for appendicular lean mass on the anabolic species count as well as combined effects from several different Mendelian randomization methods: inverse variance weighted (IVW), weighted median, penalized median, and Egger regression.
