# Supplementary material for: Identification of three bacterial species associated with increased appendicular lean mass: the HUNT study
Source: Nat Commun. 2023 Apr 20;14:2250. doi: 10.1038/s41467-023-37978-9 (PMC10119287; doi:10.1038/s41467-023-37978-9)
Supplement: Supplementary file 4 — Reporting Summary [file 41467_2023_37978_MOESM4_ESM.pdf]

nature portfolio

Corresponding author(s): Louise Grahnemo

Last updated by author(s): Mar 16, 2023

Reporting Summary

Nature Portfolio wishes to improve the reproducibility of the work that we publish. This form provides structure for consistency and transparency in reporting. For further information on Nature Portfolio policies, see our Editorial Policies and the Editorial Policy Checklist.

Statistics

For all statistical analyses, confirm that the following items are present in the figure legend, table legend, main text, or Methods section.

n/a	Confirmed	
☐	☒	The exact sample size (*n*) for each experimental group/condition, given as a discrete number and unit of measurement
☒	☐	A statement on whether measurements were taken from distinct samples or whether the same sample was measured repeatedly
☐	☒	The statistical test(s) used AND whether they are one- or two-sided<br>*Only common tests should be described solely by name; describe more complex techniques in the Methods section.*
☐	☒	A description of all covariates tested
☐	☒	A description of any assumptions or corrections, such as tests of normality and adjustment for multiple comparisons
☐	☒	A full description of the statistical parameters including central tendency (e.g. means) or other basic estimates (e.g. regression coefficient) AND variation (e.g. standard deviation) or associated estimates of uncertainty (e.g. confidence intervals)
☐	☒	For null hypothesis testing, the test statistic (e.g. *F*, *t*, *r*) with confidence intervals, effect sizes, degrees of freedom and *P* value noted<br>*Give P values as exact values whenever suitable.*
☒	☐	For Bayesian analysis, information on the choice of priors and Markov chain Monte Carlo settings
☒	☐	For hierarchical and complex designs, identification of the appropriate level for tests and full reporting of outcomes
☐	☒	Estimates of effect sizes (e.g. Cohen's *d*, Pearson's *r*), indicating how they were calculated

Our web collection on statistics for biologists contains articles on many of the points above.

Software and code

Policy information about availability of computer code

Data collection	Genomic position, strand orientation, and the reference allele of genotyped variants were determined by aligning their probe sequences against the human genome (Genome Reference Consortium Human genome (build 37) and revised Cambridge Reference Sequence of the human mitochondrial DNA (GenBank ID NC_012920); http://genome.ucsc.edu, accessed Feb 9th 2022) using BLAT (build 2016). The sequencing reads were processed using StaG-mwc workflow (v0.4.0), which conducts reads quality trimming and filtering using fastp (version 0.20.0), host reads removal using Kraken 2 (version 2.0.8_beta), and bacterial taxonomic profiling using MetaPhlAn2 (version 2.9.21). Default parameters from the workflow were used for reads processing and taxonomic classification.
Data analysis	Raw data were analyzed using R, version 4.1.1. Figures were processed in R or GraphPad Prism, version 9.1.0. Linear regressions were analyzed using the R function lm, logistic regressions using glm, and correlations using cor.test. The R package emmeans (version 1.6.1) was used to obtain estimated marginal means for groups. Microbial data was CLR transformed using the R package microbiome (version 1.19.1). The R package metafor (version 3.8-1) was used for the meta-analysis. Associations between each of the used genetic instruments and the anabolic species count were estimated with a linear mixed model adjusted for sex, age, cohort, and the first four principal components using the BOLT-LMM (version 2.3.4) software that also accounts for relatedness between samples. The MR analyses were conducted with the R-package MendelianRandomization (version 0.5.1)

For manuscripts utilizing custom algorithms or software that are central to the research but not yet described in published literature, software must be made available to editors and reviewers. We strongly encourage code deposition in a community repository (e.g. GitHub). See the Nature Portfolio guidelines for submitting code & software for further information.

Data

Policy information about availability of data

All manuscripts must include a data availability statement. This statement should provide the following information, where applicable:

- Accession codes, unique identifiers, or web links for publicly available datasets
- A description of any restrictions on data availability
- For clinical datasets or third party data, please ensure that the statement adheres to our policy

Summary statistics are presented in the article. The individual participant data are available under restricted access for privacy issues. Researchers associated with Norwegian research institutes can apply for the use of HUNT data and samples with approval by the Regional Committee for Medical and Health Research Ethics. Researchers from other countries may apply if collaborating with a Norwegian Principal Investigator. Information for data access can be found at https://www.ntnu.edu/hunt/data. The HUNT variables are available for browsing on the HUNT databank at https://hunt-db.medisin.ntnu.no/hunt-db/. The Genome Reference Consortium Human genome (build 37) and revised Cambridge Reference Sequence of the human mitochondrial DNA (GenBank ID NC_012920) were obtained from http://genome.ucsc.edu. Source data are provided with this paper.

Human research participants

Policy information about studies involving human research participants and Sex and Gender in Research.

Reporting on sex and gender	HUNT is a population-based study where gender was determined by a questionnaire. The characteristics of the study participants are provided in a disaggregated manner in Supplemental table 1, Supplemental table 8, Supplemental Table 12, and Figure 1d. Other analyses are adjusted for gender.
Population characteristics	The Trøndelag Health Study (HUNT) is a comprehensive population-based study that has collected 81 data in four surveys in the region of Nord-Trøndelag, Norway from 1984 to 2019 (HUNT1–4). Our cross-sectional study includes participants in HUNT4 (2017–19). The discovery cohort consisted of 2866 (mean age 60.3±13.9 standard deviation [SD], 59.4% women) randomly selected participants. The replication cohort consisted of 2330 (mean age 53.8±14.0 SD, 69.3% 95 women) participants.
Recruitment	All people living in the county of Nord-Trøndelag (later the two counties South and North Trøndelag have been fused into one county, Trøndelag), Mid-Norway, that would pass the age of 20 during the period the field stations were in their municipality were eligible to participate in HUNT4. Eligible participants, identified through the Norwegian National Population Register, were invited to HUNT4 by the HUNT research center, and if needed, they were reminded once.
Ethics oversight	The study was approved by the Regional Committee for Medical and Health Research Ethics in Central Norway (reference number 28052). All participants provided written informed consent. The participants did not receive any compensation.

Note that full information on the approval of the study protocol must also be provided in the manuscript.

Field-specific reporting

Please select the one below that is the best fit for your research. If you are not sure, read the appropriate sections before making your selection.

☒ Life sciences ☐ Behavioural & social sciences ☐ Ecological, evolutionary & environmental sciences

For a reference copy of the document with all sections, see nature.com/documents/nr-reporting-summary-flat.pdf

Life sciences study design

All studies must disclose on these points even when the disclosure is negative.

Sample size	No statistical method was used to predetermine sample size. Previous studies evaluating the association between gut microbes at the species level and lean mass have included 482 subjects or less and have not included replication or detailed adjustments for multiple relevant confounders. (ref 1,2 in supplementals) To identify possible reproducible associations with the primary outcome appendicular lean mass, we established a large discovery cohort (n=2866) and one replication cohort (n=2330) with in total 5196 participants.
Data exclusions	The present study included participants with appendicular lean mass data and stool microbiota data passing a quality check, samples not passing the quality check were excluded.
Replication	We used a discovery cohort for our initial analysis to find associations between lean mass and microbiota species. Significant findings was then tested in a replication cohort. As described in the manuscript, the initial screening identified five statistically significant microbial species of which three were replicated in the replication cohort.
Randomization	The discovery cohort consisted of 2866 randomly selected participants, with qPCR analyses of microbial species in one batch (batch 1). The replication cohort consisted of 2330 participants analyzed in two batches (batch 2 and 3). Batch 2 consisted of 949 selected healthy participants and 44 randomly selected participants. Batch 3 consisted of 1337 participants that were selected migraine cases (n=741) or

controls (n=596) in a migraine sub-study. We have adjusted for 1) analysis batch, 2) whether participants of batch 2 were a selected healthy subject, and 3) whether participants of batch 3 were a selected migraine case.

Blinding

Not neccessary as this is a cross-sectional population-based study with no groups.

Reporting for specific materials, systems and methods

We require information from authors about some types of materials, experimental systems and methods used in many studies. Here, indicate whether each material, system or method listed is relevant to your study. If you are not sure if a list item applies to your research, read the appropriate section before selecting a response.

Materials & experimental systems

n/a	Involved in the study	
☒	☐	Antibodies
☒	☐	Eukaryotic cell lines
☒	☐	Palaeontology and archaeology
☒	☐	Animals and other organisms
☒	☐	Clinical data
☒	☐	Dual use research of concern

Methods

n/a	Involved in the study	
☒	☐	ChIP-seq
☒	☐	Flow cytometry
☒	☐	MRI-based neuroimaging
